# Dual-inhibitory domain iCARs improve the efficiency of the AND-NOT gate CAR T strategy

**DOI:** 10.1073/pnas.2312374120

**Published:** 2023-11-14

**Authors:** Nathanael J. Bangayan, Liang Wang, Giselle Burton Sojo, Miyako Noguchi, Donghui Cheng, Lisa Ta, Donny Gunn, Zhiyuan Mao, Shiqin Liu, Qingqing Yin, Mireille Riedinger, Keyu Li, Anna M. Wu, Tanya Stoyanova, Owen N. Witte

**Affiliations:** ^a^Department of Molecular and Medical Pharmacology, University of California, Los Angeles, CA 90095; ^b^Department of Microbiology, Immunology, and Molecular Genetics, University of California, Los Angeles, CA 90095; ^c^Department of Immunology and Theranostics, Arthur Riggs Diabetes and Metabolism Research Institute, Beckman Research Institute, City of Hope, Duarte, CA 91010; ^d^Department of Molecular and Medical Pharmacology, Crump Institute for Molecular Imaging, David Geffen School of Medicine at University of California - Los Angeles, Los Angeles, CA 90095; ^e^Department of Radiation Oncology, City of Hope, Duarte, CA 91010; ^f^Department of Urology, University of California, Los Angeles, CA 90095; ^g^Jonsson Comprehensive Cancer Center, University of California, Los Angeles, CA 90095; ^h^Molecular Biology Institute, University of California, Los Angeles, CA 90095; ^i^Eli and Edythe Broad Center of Regenerative Medicine and Stem Cell Research, University of California, Los Angeles, CA 90095; ^j^Parker Institute for Cancer Immunotherapy, University of California, Los Angeles, CA 90095

**Keywords:** chimeric antigen receptor, inhibitory CAR (iCAR), on-target, off-tumor toxicity, immunotherapy, AND-NOT logic gate

## Abstract

CAR (chimeric antigen receptor) T cell therapy has shown clinical success in treating hematological malignancies, but its treatment of solid tumors has been limited. One major challenge is on-target, off-tumor toxicity, where CAR T cells also damage normal tissues that express the targeted antigen. To reduce this detrimental side-effect, Boolean-logic gates like AND-NOT gates have utilized an inhibitory CAR (iCAR) to specifically curb CAR T cell activity at selected nonmalignant tissue sites. However, the strategy seems inefficient, requiring high levels of iCAR and its target antigen for inhibition. Using a TROP2-targeting iCAR with a single PD1 inhibitory domain to inhibit a CEACAM5-targeting CAR (CEACAR), we observed that the inefficiency was due to a kinetic delay in iCAR inhibition of cytotoxicity. To improve iCAR efficiency, we modified three features of the iCAR—the avidity, the affinity, and the intracellular signaling domains. Increasing the avidity but not the affinity of the iCAR led to significant reductions in the delay. iCARs containing twelve different inhibitory signaling domains were screened for improved inhibition, and three domains (BTLA, LAIR-1, and SIGLEC-9) each suppressed CAR T function but did not enhance inhibitory kinetics. When inhibitory domains of LAIR-1 or SIGLEC-9 were combined with PD-1 into a single dual-inhibitory domain iCAR (DiCARs) and tested with the CEACAR, inhibition efficiency improved as evidenced by a significant reduction in the inhibitory delay. These data indicate that a delicate balance between CAR and iCAR signaling strength and kinetics must be achieved to regulate AND-NOT gate CAR T cell selectivity.

## Significance

On-target, off-tumor toxicity is a major barrier to the application of CAR (chimeric antigen receptor) T therapy to solid tumors. Boolean logic gates like the AND-NOT gate have utilized an inhibitory CAR (iCAR) to reduce this toxicity. We investigated the role of avidity, affinity, and internal signaling domain composition on the kinetics of iCAR inhibition. With this knowledge, we designed a dual-inhibitory domain CAR (DiCAR) that combines two immune cell inhibitory signaling domains to specifically regulate CAR T cell cytotoxicity and improve inhibition efficiency compared to an iCAR with a single PD1 domain.

Genetically engineered adoptive cell therapies that target tumor-associated antigens have recently shown success in the clinic. One such therapy is chimeric antigen receptor (CAR) T cell therapy which introduces an engineered receptor that combines an extracellular antigen binding domain and T cell signaling domains into T cells to specifically kill tumor cells. CAR T cells targeting CD19 and B-cell maturation antigen (BCMA) have successfully treated hematological malignancies such as relapsed or refractory acute lymphoblastic leukemia, large B cell lymphoma, and multiple myeloma (1, 2). Although application of CAR T cell treatment for solid tumors has rapidly grown in number of clinical trials (3), its success has been limited due to two major obstacles: the immune restrictive tumor microenvironment (4) and on-target, off-tumor toxicity (5–7). To overcome this inhibitory environment, CAR T cells have been generated to be more potent, but these improvements are still accompanied with neurotoxicity, cytokine release syndrome, and/or on-target, off-tumor toxicity (8). Improvements must be made to balance the strength and efficacy of CAR T therapy with the potential toxicities associated with it.

On-target, off-tumor toxicity occurs when CAR T cells recognize normal tissues that express the targeted tumor-associated antigen. These toxicities have ranged from manageable with the CD19 CAR and B cell aplasia (8) to lethal with the Erb-B2 Receptor Tyrosine Kinase 2 (ERBB2) CAR and respiratory distress (9). Additional CARs targeting Carcinoembryonic antigen-related cell adhesion molecule 5 (CEACAM5) in the lung and Carbonic anhydrase IX (CAIX) in the liver have also shown toxicities that were considered too debilitating to advance clinically (5–7).

Various strategies have been developed to reduce CAR T cell toxicity. Elimination of CAR T cells through drug-induced suicide genes and secondary markers (10–13), affinity tuning of the antigen binding domain (14, 15), and control of CAR T cell recognition through small molecules and targeting modules (16–19) have all been tested. Each of these strategies has been capable of reducing toxicity but at the cost of efficacy due to the loss of persistence or increased tumor escape.

Rather than compromising the efficacy of CAR T treatment, Boolean logic gates have been applied to CAR T cells as safety switches. By integrating signals from multiple receptors at once, these CAR T cells can better regulate their activity based on their environment. For example, AND-gate strategies utilize two receptors that recognize different tumor antigens to trigger CAR T cell activation. Variations of this strategy have combined a masked CAR and proteases (20), a chimeric costimulatory receptor and a first-generation CAR (21), a Synthetic Notch receptor and a CAR (22), and a logic-gated intracellular network (LINK) CAR (23). Although promising, the strategy suffers from two limitations: 1) tumor escape can occur due to loss of a single antigen and 2) leakiness of either one of the receptors can lead to toxicity (23).

An alternative logic gate, that can provide more protection, is the AND-NOT gate, which utilizes two receptors—an activating CAR that contains T cell costimulatory and activation domains and an inhibitory CAR (iCAR) that contains a T cell inhibitory signaling domain. The CAR recognizes a tumor antigen and activates a T cell, while the iCAR recognizes a normal tissue antigen and inhibits T cell activity. In this manner, the CAR T cell can distinguish a tumor cell and normal cell that express the same CAR target. Over a decade ago, Fedorov et al. published the proof of concept of this strategy by linking an scFv chain that recognized Prostate-Specific Membrane Antigen (PSMA) to the PD-1 or CTLA-4 inhibitory signaling domains. This iCAR was capable of inhibiting T cell proliferation, cytokine production, and cytotoxicity when combined with a TCR or CD19-targeting CAR. However, its ability to efficiently inhibit T cell activity was limited to when the iCAR-specific antigen was highly expressed (24).

Improvements to iCAR design have focused on targeting relevant normal tissue antigens and increasing the potency of iCAR signaling. HLA-C1, HLA-A2, and HLA-A3 have all been described as iCAR targets that limit killing to tumor cells with loss of HLA alleles (25–28), but this subjects CAR T therapy to HLA-restriction. Leukocyte Immunoglobulin Like Receptor B1 (LIR-1) and T Cell Immunoreceptor With Ig And ITIM Domains (TIGIT) have been reported as replacements to PD-1 (26, 29, 30), but how they enhance iCAR inhibition is unknown.

The AND-NOT gate strategy is compelling, but a deeper understanding of the mechanisms and key drivers of specific iCAR inhibition is necessary to achieve a tighter regulation of CAR T cell activity. Unlike CARs, the role that affinity and avidity play in iCAR function and kinetics has not been well studied. To better understand how to enhance specific iCAR inhibition of CAR T cell activity, we studied the role of dosage, affinity, and internal signaling components in iCAR inhibitory kinetics. This knowledge led us to develop a class of iCARs that combine two different inhibitory signaling domains into a single construct termed the dual-inhibitory domain iCAR (DiCAR). DiCARs more efficiently inhibit CAR T cell activity than an iCAR with a single PD1 domain.

## Results

### The TROP2-PD1 iCAR Displays a Kinetic Delay in Inhibition of CAR T Cytotoxicity.

To develop a model for the AND-NOT gating strategy, we selected two epithelial cell targets as antigens for the CAR and iCAR. CEACAM5 (**CEA**) was chosen as a CAR target because of its high expression in neuroendocrine prostate cancer (31), colorectal cancer (32), gastric cancers (33), and small cell cancers of the lung (34). Due to its normal tissue expression in the colon, bladder, kidney, and lung, some adoptive cell therapies targeting this antigen have displayed dose-limiting on-target, off-tumor toxicities that could be reduced with an AND-NOT Boolean logic gate (5, 33, 35).

As an iCAR target, we selected TROP2 *Tumor Associated Calcium Signal Transducer 2* (*TACSTD2*) or TROP2, which is widely expressed in epithelial cells of the lungs, skin, esophagus, kidney, liver, and pancreas (36). Antibody-drug conjugates targeting TROP2 have been used in the treatment of metastatic triple-negative breast cancer, making it an amenable target for immunotherapies (37). Our previous work with TROP2 made it a useful surrogate epithelial cell marker for studying the AND-NOT gating strategy (38, 39).

As an activating module, we enhanced a previously published CEACAM5-Long-CD28-3z CAR by replacing its extracellular spacer and costimulatory domain (*SI Appendix*, Fig. S1*A*) to increase in vivo functionality (31, 40–42). This CEACAM5-42NQ-41BB-3z targeting CAR (CEACAR) elicited the same levels of IFN-γ production and cytotoxicity against a CEACAM5^+^ engineered cell line as our previously published CAR (*SI Appendix*, Fig. S1 *B* and *C*) (31). This CEACAR was also able to eliminate CEACAM5^+^ tumors in vivo compared to an untransduced T cell control (*SI Appendix*, Fig. S1*D*).

**Fig. 1.**
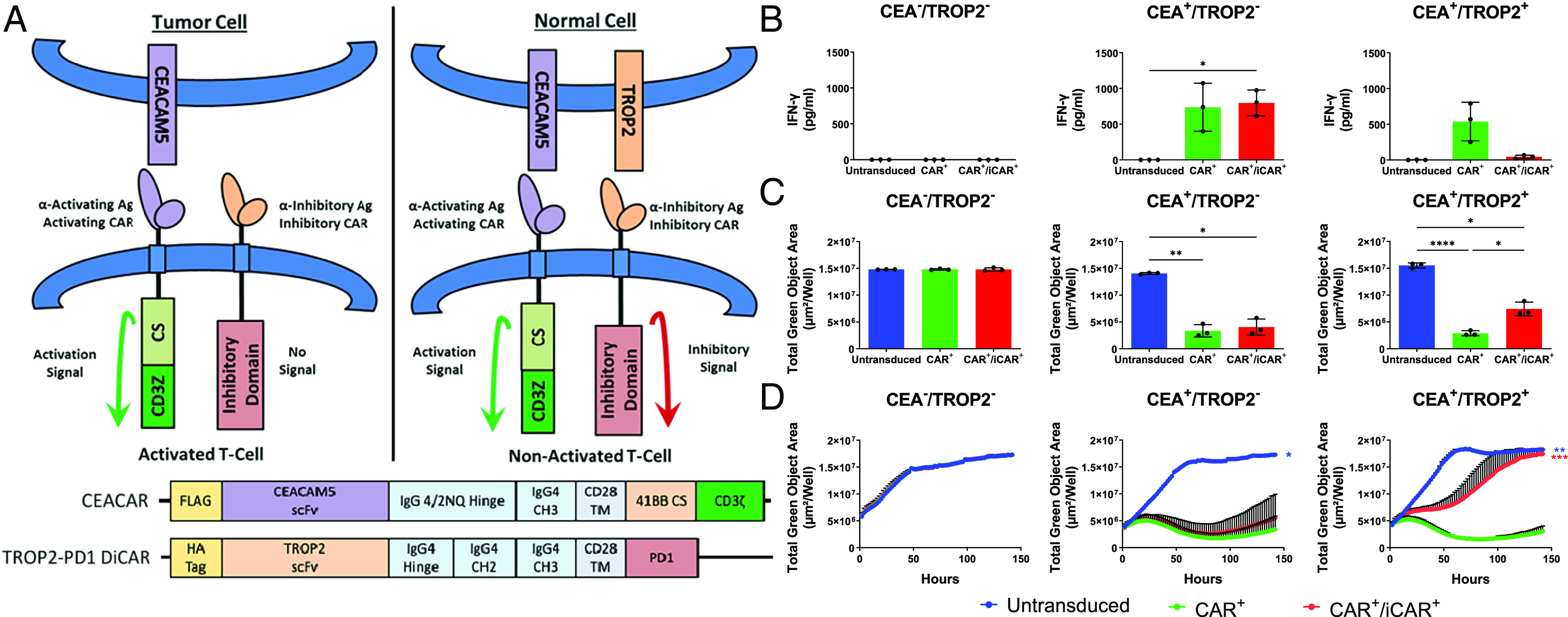
Inhibition of CAR T cell cytotoxicity by the TROP2-PD1 iCAR is delayed. (*A*) The model illustrates the “AND-NOT”-gate CAR T strategy for specifically targeting CEA^+^ tumor cells. The CAR and iCAR target CEA and TROP2, respectively. The CEACAR consists of a FLAG tag, an scFv chain that recognizes CEA, an IgG 4/2NQ hinge, an IgG4 CH3 constant domain, a CD28 transmembrane domain (TM), a 41BB costimulatory (CS) domain, and a CD3ζ activation domain. The TROP2-PD1 iCAR consists of an HA tag, an scFv chain that recognizes TROP2, the IgG4 hinge, CH2, and CH3 constant domains, a CD28 TM, and a PD1 signaling domain. (*B*) CAR^+^/iCAR^+^ T cells can inhibit CAR T cell IFN-γ production as measured by ELISA 48 h after coculture of T cells with DU145 target cells that express CEA and/or TROP2. (*C*) CAR^+^/iCAR^+^ T cells that can specifically inhibit CAR T cell cytotoxicity after 48 h in coculture with DU145 target cells that express CEA and TROP2. Target cell presence was measured by total green object area (µm^2^/well) of DU145 target cells that express CEA and/or TROP2. (*D*) Inhibition of cytotoxicity is delayed in CAR^+^/iCAR^+^ T cells when cocultured with DU145 target cells. The cytotoxicity curve shown is a composite of three donors. Measurements of total green object area of GFP+ target cells were measured over ~140 h by Incucyte live cell image analysis at intervals of 2 h. Statistics are calculated based on the total green object area (µm^2^/well) at the last time point compared to the CAR^+^ only control. The data are reported as a mean ± SE (n = 3 donors). Statistics are performed using 1-way ANOVA analysis with Tukey multiple comparison correction. *P value ≤ 0.05, **P value ≤ 0.01, and ***P value ≤ 0.001.

To develop the iCAR, antibodies were identified through phage display. Recombinant TROP2 was used as an antigen for panning a single-fold single-chain fragment variable (scFv) phage display library as further described in the Methods section (43). Using one of the antibodies (H11) with the highest binding affinity, we generated an iCAR construct as described by Fedorov et al. (24). The TROP2 scFv chain was linked to an extracellular spacer (Long Spacer—IgG4 hinge, CH2, and CH3 domain), a CD28 transmembrane domain (TM), and a PD1 intracellular signaling domain to form the TROP2-Long-PD1 iCAR (TROP2-PD1 iCAR) (Fig. 1*A*).

To establish whether the TROP2-PD1 iCAR could inhibit CEACAR T cell activity, T cells transduced with both the CAR and iCAR were cocultured with engineered DU145 prostate cancer cell lines. A DU145 cell line in which the TROP2 gene was deleted using CRISPR/Cas9n (44) (CEA^–^/TROP2^–^) was engineered to express GFP and either CEACAM5 alone (CEA^+^/TROP2^–^) or both CEACAM5 and TROP2 (CEA^+^/TROP2^+^) by lentiviral transduction. T cell activity was expected when CEACAM5 alone was expressed, but inhibition was expected when TROP2 was present as well (Fig. 1*A*). Because high levels of iCAR were reported to be necessary to inhibit allogenic T cell cytotoxicity by Fedorov et al. (24), the multiplicity of infection (MOI) of the iCAR was 10-fold higher than the CEACAR. T cells enriched to be at least 80% CAR^+^/iCAR^+^ were then cocultured with these engineered DU145 cell lines.

Two hallmarks of T cell activation that were inhibited by CAR^+^/iCAR^+^ T cells after coculture with CEA^+^/TROP2^+^ target cells were cytokine production and cytotoxicity. Approximately 90% less IFN-γ was produced by the CAR^+^/iCAR^+^ T cells compared to the CAR^+^ only control (Fig. 1*B*). This difference was not seen when the target cells expressed CEACAM5 alone. There was also a 30% reduction in the death of CEA^+^/TROP2^+^ target cells by the CAR^+^/iCAR^+^ T cells compared to the CAR^+^ only control 48 h after coculture (Fig. 1*C*)—a difference not observed with the CEA^+^/TROP2^–^ control. However, 50% of the population was still killed compared to the untransduced negative control, suggesting that the TROP2-PD1 iCAR could inhibit CEACAR cytotoxicity but not completely.

T cells rapidly integrate both positive and negative signals to determine how they will interact with a target cell. Since CAR T signaling and activity is dynamic (45), we hypothesized that this incomplete inhibition might be due to a delay in the TROP2-PD1 iCAR’s inhibitory function. To test this hypothesis, CAR^+^/iCAR^+^ T cells were cocultured with CEA^+^/TROP2^+^ target cells and observed by Incucyte live cell image analysis over 150 h (Fig. 1*D* and *SI Appendix*, Fig. S2*A*). Forty-eight hours after coculture, target cells were killed by the CAR^+^/iCAR^+^ T cells. However, at 72 h, the adherent target cells appeared to be replicating compared to those cultured with the CAR^+^ only control based on relative confluence over time. By 150 h, the target cells were confluent (*SI Appendix*, Fig. S2*A*), suggesting that inhibition had occurred. Flow cytometry analysis of target cells recovered after coculture confirmed the continued expression of CEA and TROP2, removing the possibility that the target cells survived due to CAR target antigen loss (*SI Appendix*, Fig. S2*B*). Regardless of the three independent donors used to generate the CAR^+^/iCAR^+^ T cells in replicate experiments, the TROP2-PD1 iCAR was able to inhibit CAR T cell activity, but this inhibition was delayed (Fig. 1*D*).

### Increasing iCAR Avidity Reduces the Kinetic Delay in Inhibition.

Both affinity and avidity contribute to the efficacy of antibody-based tumor targeting therapies (46). Avidity was recently shown to contribute to an iCAR’s ability to inhibit CAR NK cell activity (28). This prompted us to ask whether the delay in iCAR inhibition of T cells was also affected by its avidity. Since avidity is based on the number of receptors and antigens interacting, we investigated these variables by modulating the surface level expression of the antigens on target cells and the receptors on T cells.

**Fig. 2.**
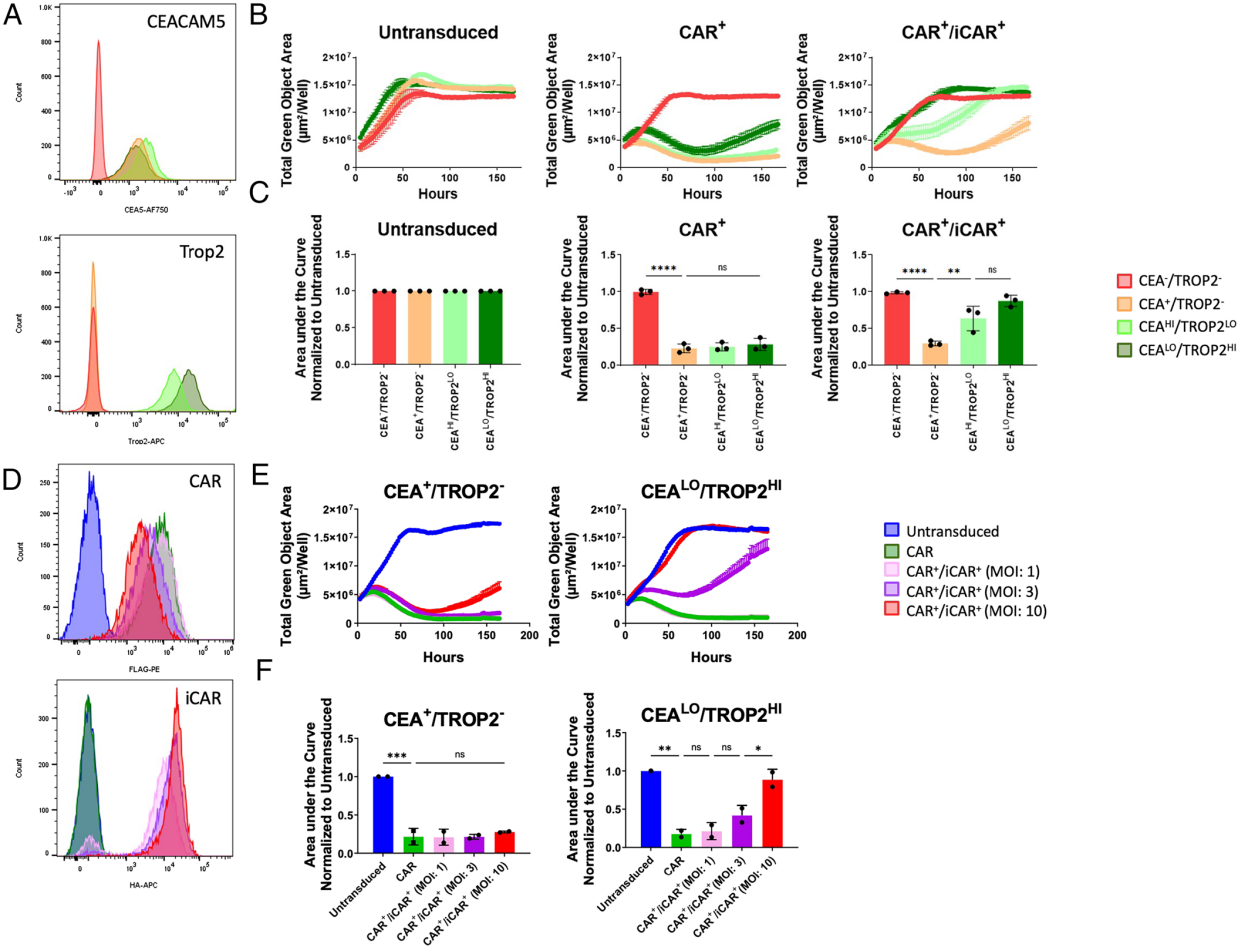
Controlling the avidity of iCAR interactions reduces the delay in inhibitory signaling kinetics. (*A*) Engineered target cell lines have different surface level expression of CEA and TROP2 measured by flow cytometry. Histograms are representative images from one of three experiments comparing CEA and TROP2 expression of each target cell line. (*B*) Increasing the target antigen density reduces the delay in iCAR inhibition as measured by cytotoxicity over time. The cytotoxicity curves are representative images from one experiment measuring the total green object area of the target cells over time (μm^2^/well). (*C*) CAR^+^/iCAR^+^ T cells cocultured with target cells that express high levels of TROP2 have reduced delays in inhibition. The delay in inhibition was measured by calculating the area under each cytotoxicity curve. The AUC was normalized against the AUC calculated for untransduced T cells cocultured with target cells. The normalized AUC quantified is the mean ± SD (n = 3). (*D*) Representative histograms measuring the Mean Fluorescence Intensity (MFI) indicate the difference in CAR and iCAR surface expression between CAR T cell groups being tested. Groups have been transduced with CAR lentivirus at an MOI of 1 and iCAR lentivirus at an MOI of 1, 3, and 10, respectively. (*E*) Increasing the surface level expression of the iCAR in primary T cells reduces the delay in iCAR inhibition as measured by cytotoxicity over time. Representative cytotoxicity curves from one experiment are displayed comparing the killing ability of CAR T cells with different surface level expression of the iCAR when cocultured with DU145 target cells that express CEA or CEA and TROP2. (*F*) CAR^+^/iCAR^+^ T cells with higher iCAR surface expression have reduced delays in inhibition when cocultured with CEA^LO^/TROP2^HI^ target cells. The delay in inhibition was measured by calculating the area under each cytotoxicity curve. The AUC was normalized against the AUC calculated for untransduced T cells cocultured with target cells. The normalized AUC quantified is the mean ± SD (n = 2). Statistics are performed using 1-way ANOVA analysis with Tukey multiple comparison correction. *P value ≤ 0.05, **P value ≤ 0.01, and ***P value ≤ 0.001.

To control the level of CAR and iCAR target antigen, the CEA^–^/TROP2^–^ target cell line was transduced with lentiviruses that contained CEA (CAR antigen) and TROP2 (iCAR antigen). These cells were single cell cloned and screened for target cell lines that had high CEA and low TROP2 expression (CEA^HI^/TROP2^LO^) and low CEA and high TROP2 expression (CEA^LO^/TROP2^HI^) (Fig. 2*A* and *SI Appendix*, Fig. S3*A*). CAR^+^/iCAR^+^ T cells were then cocultured with both these cell lines, and the delay in inhibition was compared over time. To compare the delays between groups and experiments, the area under each cytotoxicity curve (AUC) was calculated and normalized to an untransduced T cell control. The closer the normalized AUC was to 1 the more the cytotoxicity curve matched the untransduced control, suggesting a shorter delay.

When CAR^+^/iCAR^+^ T cells were cocultured with target cells that expressed higher levels of the iCAR antigen TROP2 and lower levels of the CAR antigen CEA, the delay in inhibition was reduced (CEA^LO^/TROP2^HI^ vs. CEA^HI^/TROP2^LO^, Fig. 2 *B* and *C*). The data showed that as target cells became more sensitive to the iCAR and less sensitive to the CAR, inhibition efficiency improved. Increasing the avidity by higher surface expression of the iCAR target antigen TROP2 reduced the delay in inhibition.

Another way to adjust the avidity was to alter the levels of CAR and iCAR on the surface of T cells. To increase the surface expression of the iCAR, primary T cells were transduced with increasing MOIs for the TROP2-PD1 iCAR lentivirus (MOI: 1, 3, 10), while holding the MOI of the CEACAR lentivirus constant (MOI: 1). Flow cytometry confirmed that as the MOI increased, the surface expression as measured by mean fluorescence intensity (MFI) of the iCAR increased. Concurrently, the MFI of the CAR decreased (Fig. 2*D* and *SI Appendix*, Fig. S3*B*). Overall, the iCAR:CAR ratio gradually increased as we raised the MOI of the iCAR, leading to a higher potential avidity for the iCAR.

These CAR^+^/iCAR^+^ T cells were then cocultured with the CEA^LO^/TROP2^HI^ target cell line and observed for approximately 170 h. As seen in Fig. 2 *E* and *F*, as the MOI of the iCAR increased, the efficiency of inhibition increased as measured by the cytotoxicity curves and AUCs. The CAR T cells with an iCAR at an MOI of 10 had a curve that largely overlapped with the untransduced control (Fig. 2*E*). Although we could not determine the exact iCAR:CAR ratio necessary for complete inhibition, the data suggests that efficiency of inhibition can be controlled through the avidity of the iCAR.

### Increasing the Affinity of the TROP2-PD1 iCAR Does Not Improve Efficiency of AND-NOT Gate Inhibition.

Affinities of CARs have been tuned to improve the activity and specificity of CAR T cells (14, 15, 47). Likewise, we hypothesized that we could improve the efficiency of iCAR inhibition and reduce the delay observed by increasing the affinity of the iCAR to TROP2.

**Fig. 3.**
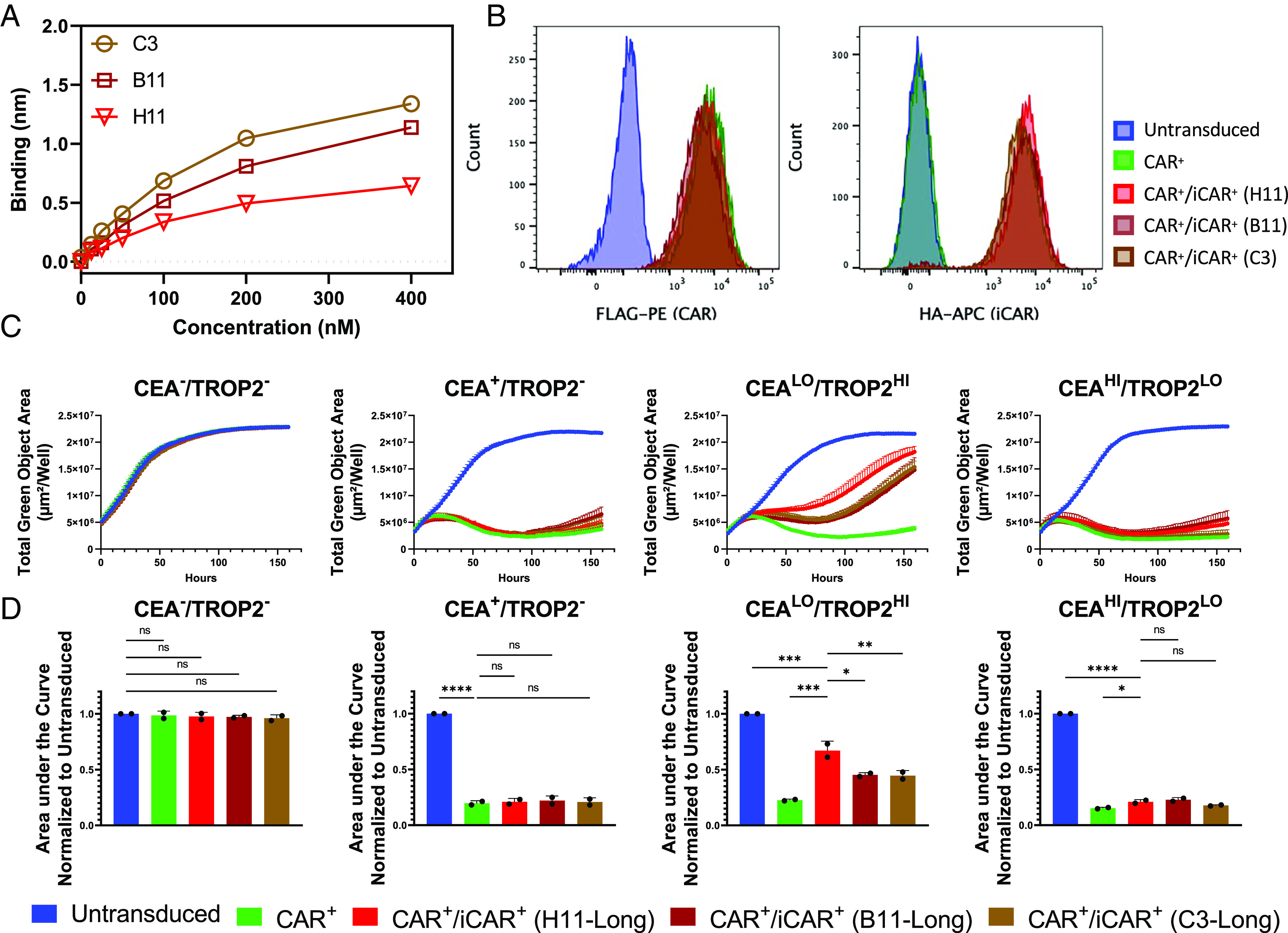
Increasing the affinity of the TROP2-targeting iCAR does not increase inhibition efficiency. (*A*) Comparison of binding affinities between three different antibodies targeting Trop2. Binding affinity kinetics were measured by BLI using biosensors precoated with recombinant TROP2 protein. Antibodies were serially diluted in concentrations ranging from 400 to 12.5 nM. The binding values were obtained and plotted against concentrations of antibody (nM). (*B*) Representative histograms from one experiment that show CAR and iCAR surface expression is similar between all T cell groups being tested. CAR and iCAR expression are measured using flow cytometry with antibodies against the FLAG- and HA-tags on the engineered receptors respectively. (*C*) CAR^+^/iCAR^+^ T cells with the C3, B11, and H11 scFv show similar levels of cytotoxicity to each other. Representative cytotoxicity curves are displayed from one experiment where the total green object area (µm/well) of GFP^+^ DU145 target cells that express CEACAM5 and/or TROP2 were measured over approximately 160 h. (*D*) Area under the curve analysis of cytotoxicity curves. The delay in inhibition was measured by calculating the area under each cytotoxicity curve. The AUC was normalized against the AUC calculated for untransduced T cells cocultured with target cells. The normalized AUC quantified is the mean ± SD (n = 2) from two independent experiments. The significance values shown are comparisons between a control group. For the CEA^–^/TROP2^–^ cell line, values are compared to the untransduced control. For the CEA^+^/TROP2^–^ cell line, values are compared to the CAR control. For the CEA^LO^/TROP2^HI^ or CEA^HI^/TROP2^LO^ cell lines, values are compared to the CAR^+^/iCAR^+^ (H11-Long) group. Statistics are performed using 1-way ANOVA analysis with Tukey multiple comparison correction. *P value ≤ 0.05, **P value ≤ 0.01, and ***P value ≤ 0.001.

Using Bio-Layer Interferometry (BLI), the C3 and B11 scFv chains in our phage display library were found to have a 4.6× and 2.6× higher binding affinity compared to the H11 clone in our original iCAR construct respectively (C3 K_d_ = 0.78 nM; B11 K_d_ = 1.37 nM; H11 K_d_ = 3.55 nM) (Fig. 3*A*). The C3 and B11 scFv chains were incorporated into iCARs by replacing the H11 scFv chain in our original TROP2-PD1 iCAR (H11-TROP2-PD1 iCAR). These three iCARs (C3-TROP2-PD1 iCAR, B11-TROP2-PD1 iCAR, and H11-TROP2-PD1 iCAR) were introduced into primary T cells with the CEACAR at an MOI of 1 for both the CAR and iCAR to ensure that avidity would not confound the results. All three iCARs were confirmed to have similar surface expression levels by flow cytometry (Fig. 3*B*). Enriched CAR^+^/iCAR^+^ T cells were cocultured with target cells and cytotoxicity observed over time.

Approximately a week after coculture, no improvement was seen in the inhibition efficiency of both higher affinity iCARs (C3-TROP2-PD1 iCAR and B11-TROP2-PD1 iCAR) (Fig. 3*C*). When calculating for normalized AUC, they were even found to be significantly worse (Fig. 3*D*). When TROP2 levels were reduced (CEA^HI^/TROP2^LO^), these iCARs showed no significant difference between each other regardless of affinity. Since the epitopes of these scFv chains were not mapped and the long spacer used may not be ideal for C3 and B11, variations of these iCARs were generated that had a short spacer (IgG4 hinge). These iCARs were less expressed on the surface of the cell compared to their long spacer counterparts and also showed worse inhibition (*SI Appendix*, Fig. S4). These results demonstrated that further increasing the affinity of our iCAR did not improve iCAR efficiency.

### iCARs with Immunoreceptor Tyrosine-Based Inhibition or Switch Motifs (ITIM/ITSM) Can Inhibit CAR T Cell Activation and Cytotoxicity.

CARs have been enhanced by replacing their costimulatory and activation domains with alternative domains (i.e., 41BB, ICOS, JAK/STAT, OX-40) that improve proliferation, cytokine production, and in vivo persistence (41, 48–50). It was recently shown that alternative inhibitory domains, such as CTLA-4, LIR-1, and TIGIT, could also replace the function of PD1 in an iCAR in T cells (24, 26, 29). Domains including KIR2DL1, LIR-1, CD300A, NKG2a, and LAIR-1 were also tested in an iCAR construct in NK cells (51).

We selected a series of inhibitory receptor signaling domains as potential modules that could inhibit CAR T cell activity. Some domains were derived from receptors that have been targeted as checkpoint inhibitors like T-Cell Immunoglobulin And Mucin Domain-Containing Protein 3 (TIM-3), CTLA-4, and Lymphocyte Activating 3 (LAG-3) (52–54). Other domains like CD5, Protocadherin 18 (PCDH18), and V-Set Immunoregulatory Receptor (VISTA) were selected due to their previous roles in T cell inhibition in mouse knock-out models (55–59). Domains from B And T Lymphocyte Associated (BTLA), Leukocyte Associated Immunoglobulin Like Receptor 1 (LAIR-1), TIGIT, Sialic Acid Binding Ig Like Lectin 7 (SIGLEC-7), and Sialic Acid Binding Ig Like Lectin 9 (SIGLEC-9) were all chosen for their inclusion of ITIM/ITSMs, which both inhibit signaling through the recruitment of phosphatases (60).

To test these domains for inhibitory function, twenty-two iCARs were constructed by linking the H11 TROP2 scFv chain, an extracellular spacer of variable length (Short or Long as described in Methods), a CD28 TM, and the intracellular domain of the inhibitory receptor as designated by Uniprot (61) (*SI Appendix*, Fig. S5*B* and *SI Appendix*, Table S1). All constructs were confirmed to be expressed on the cell surface by flow cytometry against an HA-tag on its N terminus (*SI Appendix*, Fig. S5*C*). Short spacer iCARs trafficked less effectively to the surface of the cell compared to those that contained long spacers regardless of the inhibitory signaling domain used (*SI Appendix*, Fig. S5*C*).

To rapidly screen through these iCARs, a Jurkat-NFAT-ZsGreen reporter cell line was cotransduced with both an iCAR and a CEACAM5-Long-CD28-3z CAR (iCAR MOI: 25, CAR MOI: 1) and tested for activation after coculture. When activated, these Jurkat cells increase the expression of ZsGreen and can be detected by flow cytometry, but if inhibited, they cannot (*SI Appendix*, Fig. S5*A*). Sorted CAR^+^/iCAR^+^ Jurkat cells were cocultured with target cells that expressed CEA and/or TROP2 for 24 h. Specific inhibition mediated by the iCAR was calculated by comparing the percentage of ZsGreen^+^ Jurkat cells when cocultured with CEA^+^/TROP2^–^ target cells compared to CEA^+^/TROP2^+^ cells.

Approximately 75% of CAR^+^ Jurkat cells were activated when cocultured with target cells that expressed CEACAM5 regardless of TROP2 expression. However, the TROP2-PD1 iCAR decreased the percentage of activated cells to ~40% when TROP2 was present (*SI Appendix*, Fig. S5*D*). In total, eleven additional inhibitory signaling domains were screened for their ability to inhibit CAR T cell activity. Of the twenty-two additional iCAR constructs tested, eight of them specifically inhibited CAR T cell activation when cocultured with the CEA^+^/TROP2^+^ line compared to the CEACAM5^+^ line. These eight constructs all contained an ITIM/ITSM motif (*SI Appendix*, Fig. S5*D*). The TROP2-Long-SIGLEC9 iCAR showed the greatest specific inhibition with a difference of ~40%.

iCARs containing BTLA, LAIR-1, and TIGIT inhibited CAR T cell activation even when TROP2 was not expressed. This ligand-independent inhibition may be due to tonic signaling of the iCAR at this avidity. Some non-ITIM-containing iCARs such as LAG-3 and CD5 also showed ligand-independent inhibition, but because no specific inhibition was observed, they were not further pursued.

**Fig. 4.**
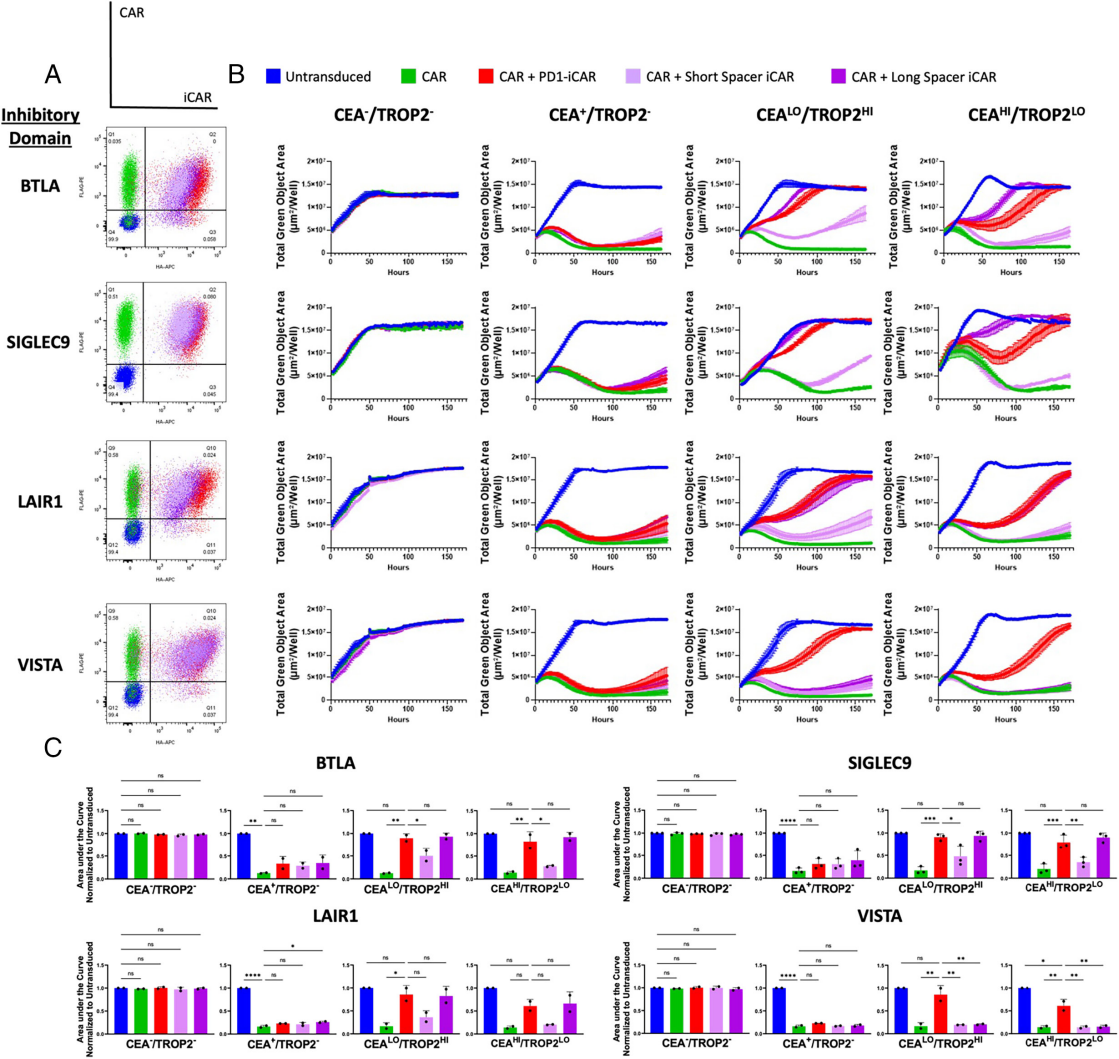
iCARs containing signaling domains with ITIM motifs can reduce CAR T cell cytotoxicity. (*A*) The five different populations (untransduced, CAR, CAR + TROP2-PD1 iCAR, CAR + TROP2-Short iCAR, CAR + TROP2-Long iCAR) tested for each inhibitory signaling domain are plotted in a flow cytometry plot that corresponds to each color in the legend. The flow cytometry plot is a representative from one experiment. (*B*) CAR^+^/iCAR^+^ T cells that were engineered with inhibitory signaling domains with an ITIM motif can inhibit CAR T cell cytotoxicity as measured by total green object area (µm/well) of DU145 target cells that express CEA and/or TROP2 over 150 h. Each curve represents a coculture with the CAR T cell population represented by the color in the legend. These cytotoxicity curves are representative images from one experiment. (*C*) CAR^+^/iCAR^+^ T cells that were engineered with inhibitory signaling domains with an ITIM motif can inhibit CAR T cell cytotoxicity with a similar efficiency as the TROP2-PD1 iCAR as measured by area under the cytotoxicity curve. AUC was normalized to the untransduced population cocultured with the target cells. The normalized AUC quantified is the mean ± SD of at least two independent experiments (BTLA—n = 2; LAIR1—n = 2, SIGLEC9—n = 3, VISTA—n = 2). The significance values shown are comparisons between a control group. For the CEA^–^/TROP2^–^ cell line, values are compared to the untransduced control. For the CEA^+^/TROP2^–^ cell line, values are compared to the CAR control. For the CEA^LO^/TROP2^HI^ or CEA^HI^/TROP2^LO^ cell lines, values are compared to the CAR + PD1 iCAR group. Statistics are performed using 1-way ANOVA analysis with Tukey multiple comparison correction. *P value ≤ 0.05, **P value ≤ 0.01, and ***P value ≤ 0.001.

A selection of iCARs (BTLA, LAIR-1, SIGLEC-9) that functioned best in the reporter assay were then tested for their ability to inhibit cytotoxicity in primary T cells equipped with the CEACAR (Fig. 4*A*). As a negative control, the VISTA iCARs were included. To lower the contribution of avidity and potential tonic signaling seen in the Jurkat reporter assay, the MOI of the iCAR was reduced to an MOI of 10. All iCARs were confirmed to be expressed on the surface of primary T cells (Fig. 4*B*).

Observing the kinetics of cytotoxicity, we found that the TROP2-Long-BTLA, LAIR-1, and SIGLEC-9 iCARs all inhibited CAR T cell cytotoxicity at a similar rate as the TROP2-PD1 iCAR when cocultured with target cells that expressed high levels of TROP2 (Fig. 4 *B* and *C*). When the TROP2 level was reduced in target cells (CEA^HI^/TROP2^LO^), the TROP2-Long-SIGLEC9 iCAR showed a reduced delay in inhibition compared to the TROP2-PD1 iCAR, suggesting that it might be more efficient.

To test whether changing the extracellular spacer length might improve the efficiency of the iCAR, we tested these same constructs with a shorter spacer length. We found that these iCARs had approximately 30 to 70% less surface expression and were less efficient at inhibiting cytotoxicity compared to their long spacer counterparts (Fig. 4 *B* and *C*). These data indicate that ITIM/ITSM-containing iCARs can inhibit CAR T cell cytotoxicity.

### DiCARs Improve iCAR Inhibitory Kinetics and Efficiency.

Third-generation CARs, which combine multiple costimulatory domains into one construct, have been reported to increase CAR T cell survival and antitumor efficacy (62–64). This led us to ask whether combining multiple inhibitory signaling domains into a single construct could further enhance inhibition efficiency.

**Fig. 5.**
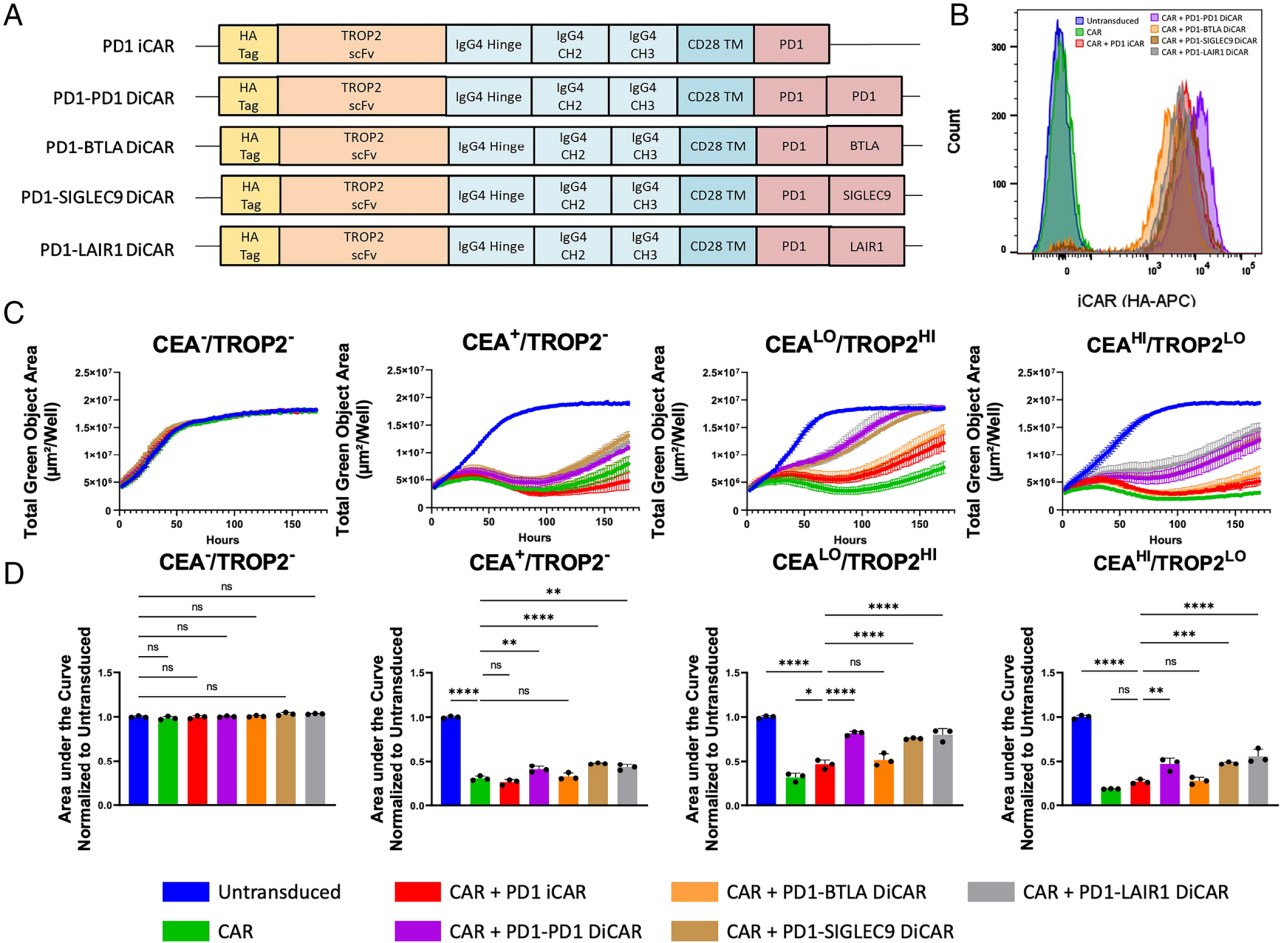
DiCARs increases the efficiency of inhibition in the AND-NOT-gate CAR T strategy. (*A*) The models represent the structure of each DiCAR tested. The DiCARs are composed of a TROP2 scFv, the IgG4 Hinge, CH2, and CH3 constant domains, a CD28 TM, the PD-1 inhibitory signaling domain, and the additional inhibitory signaling domains PD-1, BTLA, SIGLEC-9, or LAIR-1. (*B*) The representative histogram indicates that iCAR/DiCAR surface expression level is similar between the groups of DiCARs being compared. The DiCAR surface expression was determined by flow cytometry for an HA-tag located on the N terminus of the iCAR/DiCAR. (*C*) Representative cytotoxicity curves of each CAR T cell population demonstrate that CAR T cell populations with a DiCAR have a reduced delay in inhibition compared to the TROP2-PD1 iCAR. CAR T cells were cocultured with DU145 target cells that express GFP and CEACAM5 and/or TROP2. Presence of target cells was measured by total green object area (µm^2^/well) over time as measured by Incucyte live cell image analysis over 150 h. (*D*) The delay in inhibition of the iCAR was measured by area under the cytotoxicity curve analysis of each cytotoxicity curve and normalized to the coculture with the untransduced T cell group. This AUC is a representative of one experiment in which triplicate wells were analyzed. Three biological replicates were performed and reported in *SI Appendix*, Figs. S6 and S7. The significance values shown are comparisons between a control group. For the CEA^–^/TROP2^–^ cell line, values are compared to the untransduced control. For the CEA^+^/TROP2^–^ cell line, values are compared to the CAR control. For the CEA^LO^/TROP2^HI^ or CEA^HI^/TROP2^LO^ cell lines, values are compared to the CAR + PD1 iCAR group. Statistics are performed using 1-way ANOVA analysis with Tukey multiple comparison correction. *P value ≤ 0.05, **P value ≤ 0.01, and ***P value ≤ 0.001.

A series of DiCARs were designed by linking the TROP2-PD1 iCAR with an additional domain from PD-1, BTLA, SIGLEC-9, or LAIR-1 on its C terminus (Fig. 5*A*). These domains were chosen since they functioned as a single-domain iCAR. Only the long extracellular spacer was incorporated into the DiCARs because short spacer constructs were consistently shown to be less efficient as single-domain iCARs (Fig. 4 *B* and *C*).

Primary T cells were transduced with the CEACAR at an MOI of 1 and the DiCAR at an MOI of 1 to further reduce the contribution of avidity to inhibition. All DiCARs were confirmed to traverse to the cell surface as detected by flow cytometry (Fig. 5*B*). DiCAR surface expression was similar between all constructs except for the PD1-BTLA DiCAR, which always had the lowest expression and transduction efficiency (*SI Appendix*, Fig. S6). Enriched CAR^+^/iCAR^+^ T cells (>94%) were cocultured with target cells that expressed CEACAM5 and/or TROP2 and monitored for cytotoxicity over a week to observe the delay in inhibition. Three DiCARs (PD1-PD1, PD1-SIGLEC9, and PD1-LAIR1) inhibited CAR T cell cytotoxicity more efficiently than the TROP2-PD1 iCAR as indicated by a faster recovery of target cells (Fig. 5*C*). The delay in inhibition was significantly decreased as calculated by AUC (Fig. 5*D*) regardless of high or low TROP2 expression. This trend of improved inhibition by DiCARs was found to be reproducible in three independent experiments although the quantitative effect varied (*SI Appendix*, Fig. S7).

## Discussion

### Epithelial Cell Markers Like TROP2 Can Be used As iCAR Targets for AND-NOT Gating Strategies.

By combining a CD-19 targeting CAR and a PSMA targeting iCAR, Fedorov et al. showed that an AND-NOT gating strategy could potentially solve the on-target, off-tumor toxicity problem of CAR T cells (24). To make it clinically applicable, many groups began to target Human Leukocyte Antigen (HLA) molecules with the iCAR. Because HLA is expressed on most normal tissues but down-regulated by tumor cells, this target could provide broad protection (25–28, 30). However, by using HLA-directed iCARs, CAR T therapy becomes subject to HLA-restriction, circumventing a key benefit it provided over TCR-based immunotherapies. Additionally, HLA-directed iCARs can lead to inhibition of proliferation during the production of CAR^+^/iCAR^+^ T cells since HLA is expressed on T cells.

Here, we report that a CAR can also be combined with an iCAR targeting a normal epithelial cell marker like TROP2. Although TROP2 is highly expressed in tumors of epithelial cell origin (65) and has been championed as a CAR target (66), in the correct context, it can be used as an iCAR target as well. TROP2 is widely expressed in normal tissues of the kidneys, lung, and skin, and if matched with a CAR that targets cancers without TROP2 expression can provide protection without HLA-restriction (36). Future work targeting other broadly expressed epithelial cell markers like EpCAM (67), E-Cadherin (68), and Claudin-4 (69) could also be promising.

### Balancing the Levels of CAR and iCAR Signaling is Critical to Obtaining Specific Inhibition.

While testing the TROP2-PD1 iCAR for specific inhibition against the CEACAR, we observed a delay in its ability to inhibit cytotoxicity. This delay was found to be avidity dependent and correlated to the iCAR:CAR ratio. This result may explain why in previous studies with both T cells and NK cells, iCAR inhibition was enhanced with its overexpression (24, 28). Interestingly, as the amount of iCAR increased, the level of ligand independent inhibition also increased (Fig. 2*E*). Our data suggest that a balance between the number of CARs and iCARs signaling is critical to obtain specific inhibition. Accurate quantification of CAR and iCAR expression is necessary to determine a therapeutic window for this strategy.

### Increasing the Affinity of the iCAR Did Not Enhance Inhibition Efficiency.

As an alternative to balancing the ratio of CAR and iCAR, we sought to build a more efficient iCAR. We first sought to increase the affinity of the iCAR scFv chain by three- to five-fold (Fig. 3). Although enhancing iCAR affinity was expected to increase function, it did not. Though surprising, this result is not unprecedented in CAR engineering. It has been reported that if CARs reach an affinity threshold further enhancement does not improve activity (47, 70). Because all three scFv chains tested were of “high” affinity, we might have already reached that threshold. Differences in affinity of 10- to 20-fold may be required to see significant changes.

It is unclear as to why the H11-TROP2-PD1 iCAR seemed to function better than the C3 and B11 ones which had higher binding affinities when TROP2 levels were high (Fig. 3). One hypothesis was that the spacer length for both the C3 and B11 antibodies were not optimized for binding their corresponding epitopes. To address this possibility, we changed the length of the spacer in *SI Appendix*, Fig. S4. However, this modification further reduced the efficiency, suggesting that another variable or combination of variables might be contributing to this difference.

### ITSM But Not Non-ITIM/ITSM Inhibitory Domains Improve iCAR Efficiency.

A second change made to potentially increase iCAR inhibition efficiency was to replace the PD-1 domain with a non-ITIM/ITSM containing domain like LAG-3, TIM-3, or CTLA-4. None of the seven domains, including CTLA-4 which was reported by Fedorov et al. (24) to function, were capable of specifically inhibiting activity in our Jurkat activation screen (*SI Appendix*, Fig. S5). Although thirteen constructs were evaluated, the potential combinations of spacer/hinge, transmembrane domain, and signaling domain were not exhausted. Because spacers and transmembranes are known to affect CAR function (71, 72), we cannot exclude the possibility that inhibition could have been seen if another construct was used.

It is unclear as to why intracellular signaling domains from known checkpoint inhibitors like LAG-3 and TIM-3 did not specifically inhibit CAR T cell activation in this assay. Alternative inhibitory mechanisms utilized by these non-ITIM-containing inhibitory receptors may be unable to inhibit CAR T cell activation. LAG-3 functions through its KIEELE and FxxL motif, but its mode of inhibition is unknown (73, 74). TIM-3 is thought to function by either destabilizing the immunological synapse through the recruitment of phosphatases or recruiting FYN and CSK to the membrane to inactivate Lck (75, 76). It may be that SHP-1 and/or SHP-2 phosphatases that are recruited via the ITIM motif are necessary for CAR inhibition.

This concept is further strengthened by the fact that the domains that have been shown capable of replacing PD-1 in an iCAR by other groups and ours all contain ITIM/ITSM motifs. The LIR-1 domain described by Hamburger et al. contains four ITIM motifs (26), while all the NK receptor domains tested by Li et al. (KIR2DL1, LIR-1, CD300a, NKG2A, and LAIR-1) all contain varying numbers of ITIM or ITIM-like motifs (51). Because these motifs are important for the recruitment of the phosphatases SHP-1 and/or SHP-2, which dephosphorylate T cell activation proteins like Zap-70 and LAT (60), the number of ITIMs may correlate to iCAR inhibition efficiency. This may explain why the PD1-LAIR1 DiCAR can perform at similar efficiencies as the PD1-PD1 and PD1-SIGLEC9 DiCARs although it has lower surface expression than the PD1-PD1 and PD1-SIGLEC9 DiCARs (Fig. 5 and *SI Appendix*, Fig. S6). In total, the PD1-LAIR1 DiCAR would have three ITIMs and one ITSM, while the PD1-BTLA, PD1-PD1, and PD1-SIGLEC9 DiCARs would all have two ITIMs with varying numbers of ITSM or ITIM-like domains (60, 77, 78). By having one additional ITIM, the PD1-LAIR1 DiCAR may recruit more phosphatases to the membrane, increase dephosphorylation, and more rapidly inhibit CAR T cell activation even with lower numbers of receptors on the cell surface. Furthermore, the LAIR-1 domain has been found to be constitutively associated with the phosphatase SHP-1 (79) and could increase the kinetics of inhibition. Alternatively, since these phosphatases bind ITIM domains via an SH2 domain that can affect their activation (80, 81), as well as proximity to CARs, the geometry of ITIMs in these DiCARs may contribute to its inhibition efficiency.

Future work should be focused on two major aspects of enhancing this AND-NOT gate design. First, efforts must be concentrated on determining which combinations of spacers, transmembrane domains, and inhibitory domains can be combined to generate DiCARs with enhanced specificity and reduced ligand-independent inhibition. The combination of domains assessed in DiCARs here was not exhaustive, and additional constructs may further enhance the dynamic range of this strategy.

Second, experiments should be performed to determine the in vivo specificity of CAR^+^, DiCAR^+^ T cells. For these in vivo studies, a replacement pair of CAR and DiCAR antigens that are clinically relevant should be investigated. These antigens should match the following criteria: 1) the CAR antigen should have low expression in normal tissues, 2) the DiCAR antigen should have high expression in normal tissues that express the CAR antigen, and 3) the DiCAR antigen should be stably expressed on the surface of the cell, ubiquitously expressed in all normal tissues where on-target, off-tumor toxicity would be anticipated, and not be prone to cleavage. The TROP2 antigen selected in this study is suspected to be cleaved in vivo by proteases like ADAM17 (39), matriptase (82), and/or ADAM10 (83), which may explain why in preliminary studies we have found reduced expression of this antigen. Optimization of CAR dosage, DiCAR dosage, T cells injected, and antigen expression in tumor cells will need to be determined and optimized to achieve tumor elimination with reduced toxicity in vivo and are currently underway.

Just as second-generation CARs combined a costimulatory domain with the activation domain to enhance CAR T cell function, the DiCARs presented here combine two inhibitory domains to become a second-generation iCAR. The AND-NOT gating strategy can be applied to reduce on-target, off-tumor toxicity by balancing the enhanced strength of CARs with the better regulation of DiCARs.

## Materials and Methods

### Cell Line Generation.

The DU145 prostate cancer target cell line was previously modified to knock-out TROP2 expression (CEA^–^/TROP2^–^) using a CRISPR-Cas9 strategy (44). To generate target lines that express CEA and/or TROP2, CEA and TROP2 were cloned into separate lentiviral constructs and transduced into the CEA^-^/TROP2^-^ cell line. Each cell line was also engineered to express GFP for cytotoxicity assays. Following transduction, cells were single cell sorted for CEA, TROP2, and/or GFP expression. Clones were selected that had the desired surface expression of CEA and/or TROP2. Surface expression of CEA and TROP2 was confirmed by flow cytometry using the antibodies listed in *SI Appendix*, Table S2. The Jurkat-NFAT-ZsGreen reporter cell line was a gift generated and given by Dr. David Baltimore’s lab.

### Lentivirus Production.

Lentivirus for the various CARs and iCARs were generated using a previously published protocol (84). Briefly, 293T cells were grown in Dulbecco's Modified Eagle Medium (DMEM) + 10% Fetal Bovine Serum (FBS). 293T cells were transfected with Mirus TransIT 293 (Mirus, MIR2705). One day after transfection, cells were treated with 10 mM sodium butyrate for 6 to 8 h. Media were replaced with collection media (UltraCULTURE /Pro293-AM + GlutaMAX + 20 mM HEPES). Two days later, viral supernatant was collected, filtered through a 0.45-µM filter, and concentrated using Amicon Ultra-15 (100,000 NMWL) filters (Millipore, UFC910024). Virus was frozen and titered on 293T cells.

### Identification of TROP2 Binding Antibodies Using Phage Display.

A human scFv phage display library previously published by Li et al. was used to identify antibodies binding TROP2 (43). The phage library was panned with recombinant TROP2 extracellular domain-Fc chimera (R&D Systems, 650-T2-10). Clones that bound TROP2 were found using an anti-M13 antibody that recognizes the phage by ELISA. Complete antibody molecules (scFv-Fc) were generated by linking the scFv to human IgG1 Fc on the C terminus and cloned into an expression vector. Stable transfectants for antibody production were generated using Zeocin selection. The supernatant from these transfections was collected, filtered, purified, and concentrated to yield a concentration of 0.1 to 1 mg/mL. These antibodies were confirmed to specifically bind TROP2 by flow cytometry against an engineered TROP2^+^ cell line. Binding kinetics of each antibody were determined using BLI. Recombinant TROP2 extracellular domain protein was bound to the sensor surface and anti-TROP2 antibodies added in concentrations ranging from 400 to 12.5 nM. Binding affinity was calculated using FortéBio Data Analysis software. Sequences of the desired scFv’s were then utilized as the antigen-binding domain of iCARs.

### CAR and iCAR Vector Construction.

The CEACAM5 CAR was previously designed and produced by combining the CEACAM5-targeting scFv (Labetuzumab) (85), an IgG4 hinge, the IgG4 CH2 and CH3 constant domains, a CD28 transmembrane domain, a CD28 costimulatory domain, and a CD3ζ activation domain (31). Modifications to the CEACAM5 CAR were made to replace the spacer region (IgG4 Hinge + CH2 + CH3) with a spacer developed by Hudecek et al., which we termed the 4/2NQ spacer (40). Additional changes were made to replace the CD28 costimulatory domain with the 41BB costimulatory domain to generate the CAR used throughout this paper (CEACAR). iCARs were generated using a similar structure to that previously published (24). Antibodies that react to TROP2 as identified by screening a phage display library were converted into scFv chains. The scFv chain was linked to various extracellular spacers (Short—IgG4 Hinge; Long—IgG4 Hinge + CH2 + CH3), the CD28 transmembrane domain, and a series of intracellular signaling domains from immune cell inhibitory receptors. The exact amino acids that were used for the intracellular signaling domains are listed in *SI Appendix*, Table S1. DiCARs are generated by linking an anti-TROP2 scFv chain to an extracellular spacer, a CD28 transmembrane domain, a PD-1 signaling domain as listed in *SI Appendix*, Table S1, and an additional signaling domain (i.e., PD-1, BTLA, SIGLEC-9, LAIR-1) as listed in *SI Appendix*, Table S1. Both the CAR and iCAR were cloned into a third-generation lentiviral vector pCCL-c-MNDU3 generously given by Dr. Gay Crooks and Dr. Donald Kohn.

### Primary CAR T Cell Generation, Enrichment, and Characterization.

Peripheral blood mononuclear cells (PBMCs) were purchased from All Cells, LLC from various donors. Unless stated otherwise, in each experiment, a single donor was used for all groups being compared to remove donor variability within the experiment. T cells and PBMCs were grown in T Cell Media (TCM) Base supplemented with the listed cytokines (TCM Base = AIM-V Media (Thermo Fisher, 12055) supplemented with 5% human heat-inactivated AB serum (Omega Scientific, HS-25), GlutaMAX (Thermo Fisher, 35050-061), and 55 µM of Beta-mercaptoethanol). PBMCs were initially thawed and cultured in TCM Base + 50 U/mL IL-2 (PeproTech, 200-02). PBMCs were activated with Human T-Activator CD3/CD28 Dynabeads (Thermo Fisher, 11132D) at a 1:1 cell:bead ratio and plated overnight at 37 °C at a concentration of 1 × 10^6 cells/mL. The following day activated cells with beads were collected and resuspended in fresh TCM + 50 U/mL IL-2 and diluted to a concentration of 0.5 × 10^6 cells/mL and plated into a 24-well plate. Cells were transduced with lentivirus containing the iCAR at the appropriate MOI of 1, 3, or 10. Infections were supplemented with Protamine Sulfate at a concentration of 100 µg/mL. Six hours after incubation with the iCAR lentivirus, the supernatant was removed, and CAR lentivirus was added with fresh Protamine Sulfate. The next day, an additional 1 mL of media was added to each well. Seven days after activation, Dynabeads were removed, and T cells were transferred to TCM Base + 50 U/mL IL-2 + 0.5 ng/mL IL-15 (PeproTech, 200-15) media at a concentration of 1 × 10^6 cells/mL. On day 9, T cells were enriched for CAR+, iCAR+ T cells using magnetic bead enrichment. Briefly, CAR^+^ T cells were selected after staining with an Anti-FLAG-PE antibody and enriched using the EasySep Release Human PE Positive Selection Kit (Stemcell, 17654) since CARs were linked to a FLAG-tag on their N-terminal end. These cells were then selected for iCAR^+^ T cells by staining with an Anti-HA-APC antibody and enriched using the EasySep APC Positive Selection Kit (Stemcell, 17681) since iCARs were linked to an HA-tag on their N-terminal end. On day 11, magnetic beads used for enrichment were removed. On day 12, T cells were characterized by flow cytometry and used for various cytotoxicity assays. For ELISAs, 48 h after coculture began, the supernatant was harvested from each well. The supernatant was used to measure IFN-γ using the BD OptEIA Human IFN-γ Set (BD, 555142).

### Jurkat Activation Reporter Assay.

To rapidly screen an iCAR’s potential to inhibit CAR T cell activity, a Jurkat reporter assay was utilized. The Jurkat-NFAT-ZsGreen reporter cell line was generously provided by Dr. David Baltimore. These cells were transduced with a lentivirus containing the CEACAM5-Long-CD28-3z CAR previously published by our lab at an MOI of 1 (31). CAR^+^ Jurkat cells were also transduced with a lentivirus containing the selected iCAR at an MOI of 25. CAR^+^/iCAR^+^ Jurkat cells were sorted and used in a coculture assay. Jurkat cells were incubated with DU145 target cells for 24 h at an effector:target ratio of 1:1 in Roswell Park Memorial Institute media (RPMI) + 10% FBS + Glutamine (RPMI10+). Jurkat cells were then collected from the culture, and the percentage of ZsGreen+ cells was measured by flow cytometry. Gating was performed on CD3+ cells to ensure that GFP+ target cells were not contributing to the measurement.

### T Cell Kinetic Cytotoxicity Assay.

Plates are coated with 0.001% Poly-L-Lysine for at least 30 min at 37 °C. DU145 target cells that are GFP+ are collected from culture and plated in RPMI10+ (RPMI + 10% FBS + 40 mM glutamine) at the desired concentration into the coated plate. Effector CAR T cells are collected from culture and washed with 1× PBS. CAR T cells are counted and plated at the desired concentration in RPMI10+ into wells that contain target cells. Cocultures are performed at the effector:target ratio described in the figures. Cocultures are imaged using an Incucyte Zoom Live Cell Analysis System (Sartorius) over a week at approximately 2-h intervals. Masking is performed to calculate the area covered by GFP+ target cells. AUC analysis is performed using GraphPad Prism over time.

### Flow Cytometry Analysis.

Cells are collected from culture and washed with 1× Phosphate Buffered Saline (PBS). Cells are stained with the selected antibodies in Fluorescence-Activated Cell Sorting (FACS) buffer (1× PBS + 3% fetal bovine serum + 0.09% sodium azide). Antibodies that were used are listed in *SI Appendix*, Table S2. After staining, cells are washed with 1× PBS and resuspended in FACS Buffer. Cells are run on the BD FACS Canto, the BD FACSAria, or the HT LSR II. Quantification of the amount of CAR and iCAR surface expression was performed using Quantum Simply Cellular anti-Mouse IgG (Bangs Laboratories, 815A) and anti-Rat IgG beads (Bangs Laboratories, 817A) using geometric MFI.

### Xenograft Model for CEACAR Tumor Killing.

Animal experiments were conducted according to a protocol approved by the Division of Laboratory Medicine at the University of California, Los Angeles. NSG mice were obtained from The Jackson Laboratory at 68 wk of age. Engineered DU145 lines that express CEACAM5 and/or TROP2, GFP, and YFP-Luciferase were mixed with Matrigel Matrix Basement Membrane (Corning 354234) and engrafted into mice subcutaneously on the right flank. T cells were prepared as described in Primary CAR T cell Generation, Enrichment, and Characterization. Approximately, 3 wk after engraftment, when tumors were measurable (10 to 100 mm^3^), 2 × 10^6^ or 4 × 10^6^ T cells were injected into mice via tail vein. Weekly caliper measurements were obtained of the tumors starting the second week after T cell injection.

## Supplementary Material

Appendix 01 (PDF)

## Data Availability

All study data are included in the article and/or *SI Appendix*.
