# Supplementary material for: Dual-inhibitory domain iCARs improve the efficiency of the AND-NOT gate CAR T strategy
Source: Proc Natl Acad Sci U S A. 2023 Nov 14;120(47):e2312374120. doi: 10.1073/pnas.2312374120 (PMC10666036; doi:10.1073/pnas.2312374120)
Supplement: Supplementary file 1 — Appendix 01 (PDF) [file pnas.2312374120.sapp.pdf]

PNAS

Supporting Information for

Dual inhibitory domain iCARs improve efficiency of the AND-NOT gate CAR T strategy

Nathanael J. Bangayan, Liang Wang, Giselle Burton Sojo, Miyako Noguchi, Donghui Cheng, Lisa Ta, Donny Gunn, Zhiyuan Mao, Shiqin Liu, Qingqing Yin, Mireille Riedinger, Keyu Li, Anna M. Wu, Tanya Stoyanova, Owen N. Witte

Corresponding Author: Nathanael J. Bangayan and Owen N. Witte

Email: N.J.B. (nbangayan@mednet.ucla.edu) and O.N.W. (owenwitte@mednet.ucla.edu)

This PDF file includes:

Figures S1 to S7

Tables S1 to S2

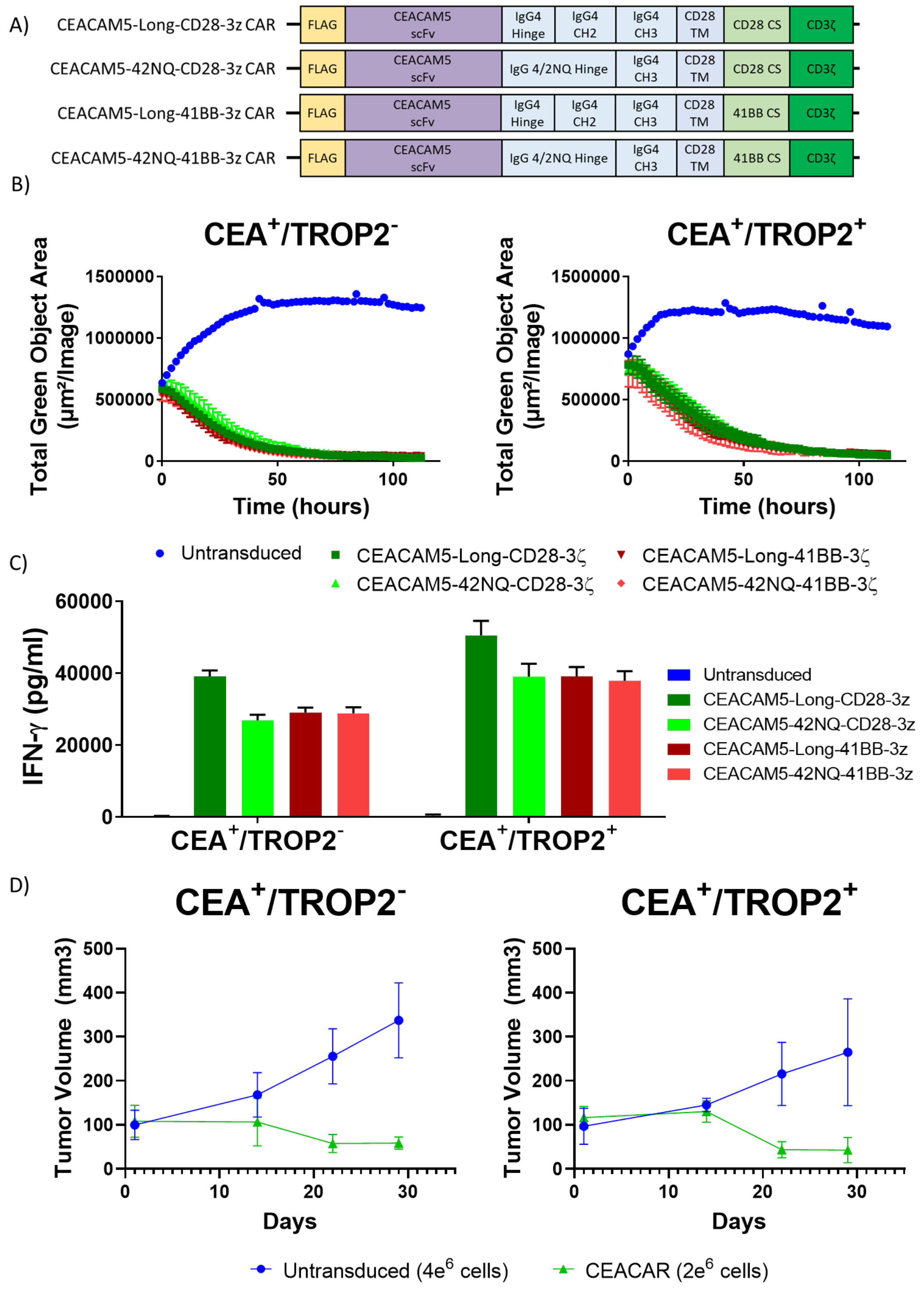

Fig. S1. CEACAM5 CARs with the natural or modified IgG4 hinge and different co-stimulatory domains have comparable cytotoxicity and IFN-γ production.

A) The illustration depicts the different CAR structures being tested. All constructs use the same scFv chain that recognizes CEACAM5. They differ in the hinge that is used. The 4/2NQ modified hinge is derived from Hudecek *et al.* (1) which combines the hinge of IgG4 and IgG2.

The IgG4 CH3 constant domain is used in all constructs. Another difference is the costimulatory domain which is derived from CD28 or 41BB. TM = Transmembrane Domain; CS = Co-stimulatory Domain.

B) All CEACAM5 CARs show comparable cytotoxicity of DU145 target cells that express CEACAM5 as measured by Incucyte live cell image analysis of total green object area of target cells (μm^2/image) over time.

C) All CEACAM5 CARs show comparable production of IFN-γ as measured by ELISA 48 hours after CAR T cells are co-cultured with DU145 target cells that express CEA or CEA and TROP2.

D) CEACAR T cells can kill $CEA5^+$ tumors *in vivo*. Mice were engrafted with either a $CEA5^+/TROP2^-$ OR $CEA5^+/TROP2^+$ tumor. Mice were injected with $4e^6$ untransduced or $2e^6$ CEACAR T cells. Caliper measurements of tumors were measured weekly two weeks after injection of T cells. n = 4 or 5 mice/group.

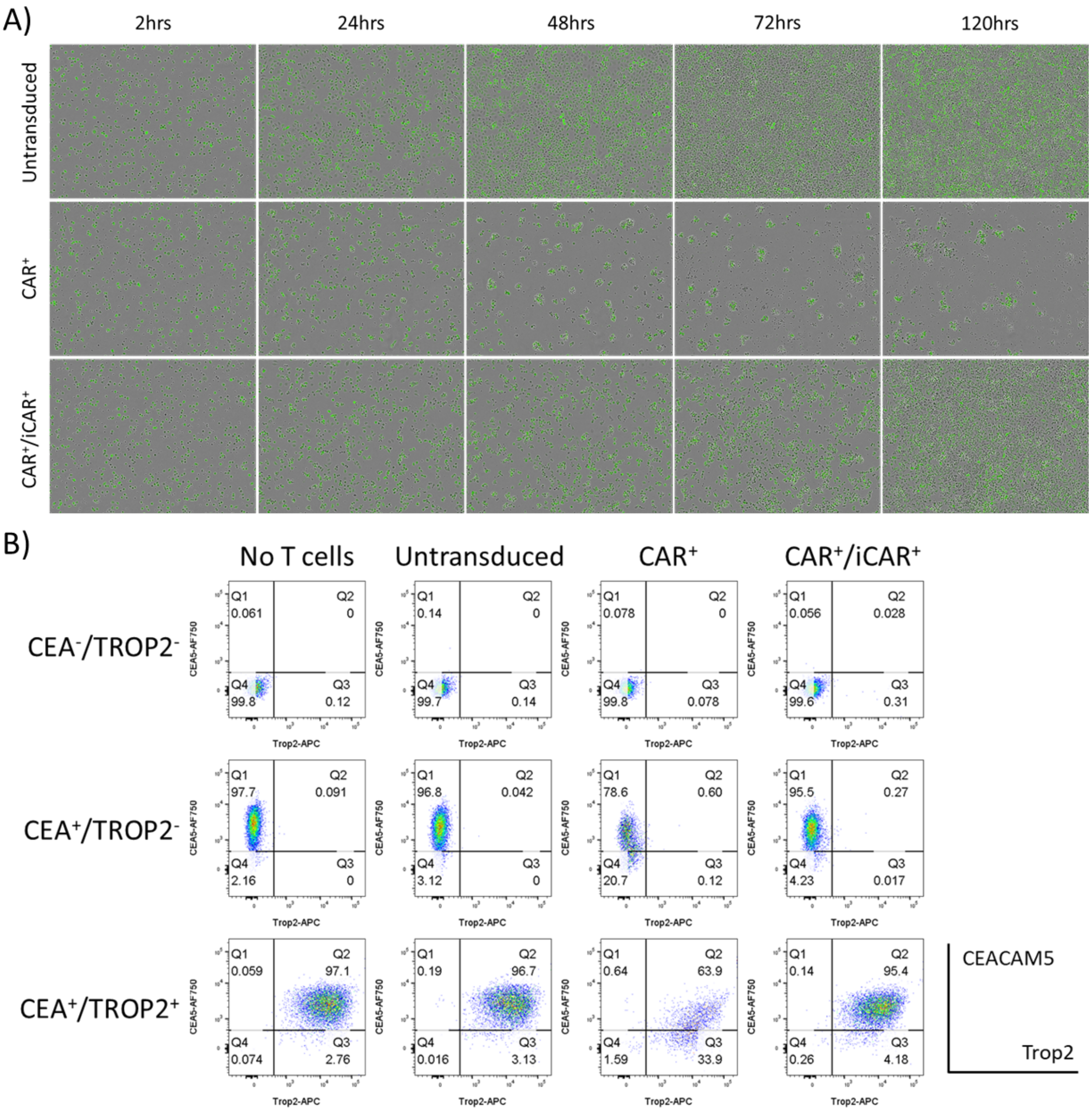

Fig. S2. CEA$^+$/TROP2$^+$ cells regrow after co-culture with CAR$^+$/iCAR$^+$ T cells

A) Images of CEA$^+$/TROP2$^+$ DU145 target cells at 2, 24, 48, 72, and 120 hours after co-culture with untransduced, CAR$^+$, or CAR$^+$/iCAR$^+$ T cells.

B) Target cells continue to express CEACAM5 and TROP2 after co-culture with CAR$^+$/iCAR$^+$ T cells. In a separate experiment, nine days after co-culture, target cells were harvested from wells where DU145 target cells that expressed CEACAM5 and/or TROP2 were co-cultured with untransduced, CAR$^+$, or CAR$^+$/iCAR$^+$ T cells. Expression of CEACAM5 and TROP2 after co-culture was measured by flow cytometry using Anti-CEACAM5 and Anti-TROP2 antibodies.

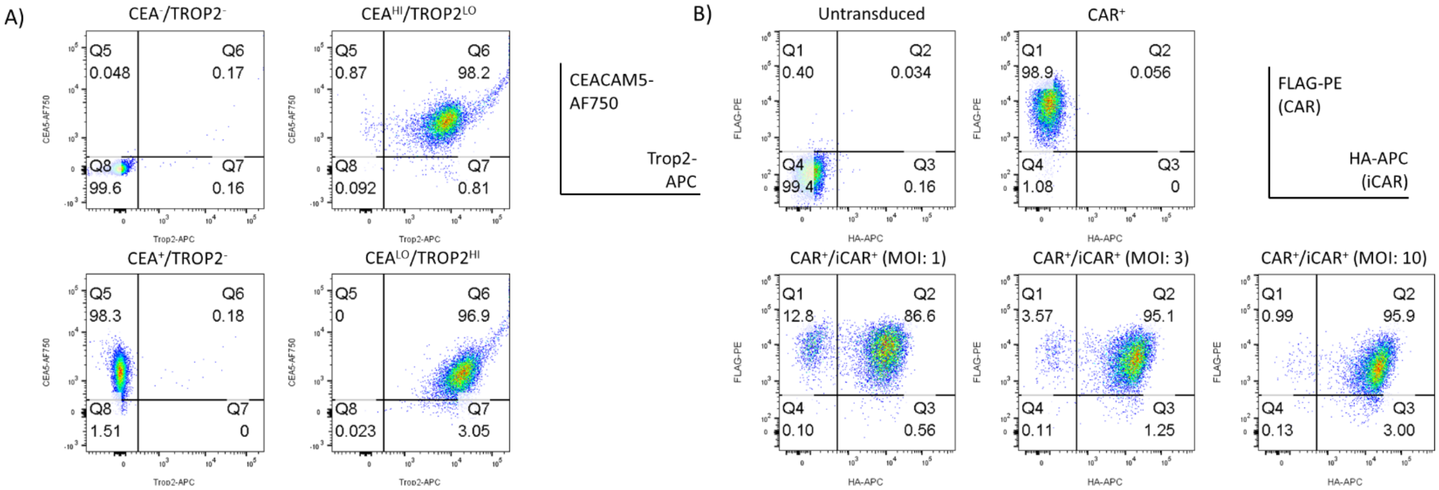

Target Cell Line	Average Geometric Mean Fluorescenc Intensity CEACAM5-AF750	Average Geometric Mean Fluorescenc Intensity Trop2-APC
CEA$^-$/TROP2$^-$	43 ± 16	0 ± 26
CEA$^+$/TROP2$^-$	1485 ± 196	11 ± 21
CEA^{HI}/TROP2^{LO}	2010 ± 494	7368 ± 3440
CEA^{LO}/TROP2^{HI}	1152 ± 136	17810 ± 3534

T Cells	Average Geometric Mean Fluorescenc Intensity FLAG-PE	Average Geometric Mean Flourescence Intensity HA-APC
Untransduced	101 ± 0	76 ± 23
CAR	6429 ± 676	72 ± 22
CAR$^+$/iCAR$^+$ (MOI: 1)	5766 ± 1030	8947 ± 346
CAR$^+$/iCAR$^+$ (MOI: 3)	4056 ± 405	14898 ± 1002
CAR$^+$/iCAR$^+$ (MOI: 10)	3033 ± 971	19099 ± 161

Fig. S3. Quantitative analysis of antigen expression in target cells and receptor expression in T cells

A) Engineered DU145 target cell lines have different surface level expression of CEA and TROP2. The flow cytometry plots are representative images from one of three experiments comparing the CEA and TROP2 expression of each target cell line. The average mean fluorescence intensity and standard deviation from three experiments are reported in the table below.

B) Engineered CAR T cells express both CAR and iCAR on the surface of the cells. Representative flow cytometry plots depict surface level expression of CAR and iCAR in the populations. Primary T cells were transduced with lentivirus containing the CAR at a MOI of 1 and lentivirus containing the iCAR at a MOI of 1, 3, and 10. The average mean fluorescence intensity and standard deviation from two experiments are reported in the table below.

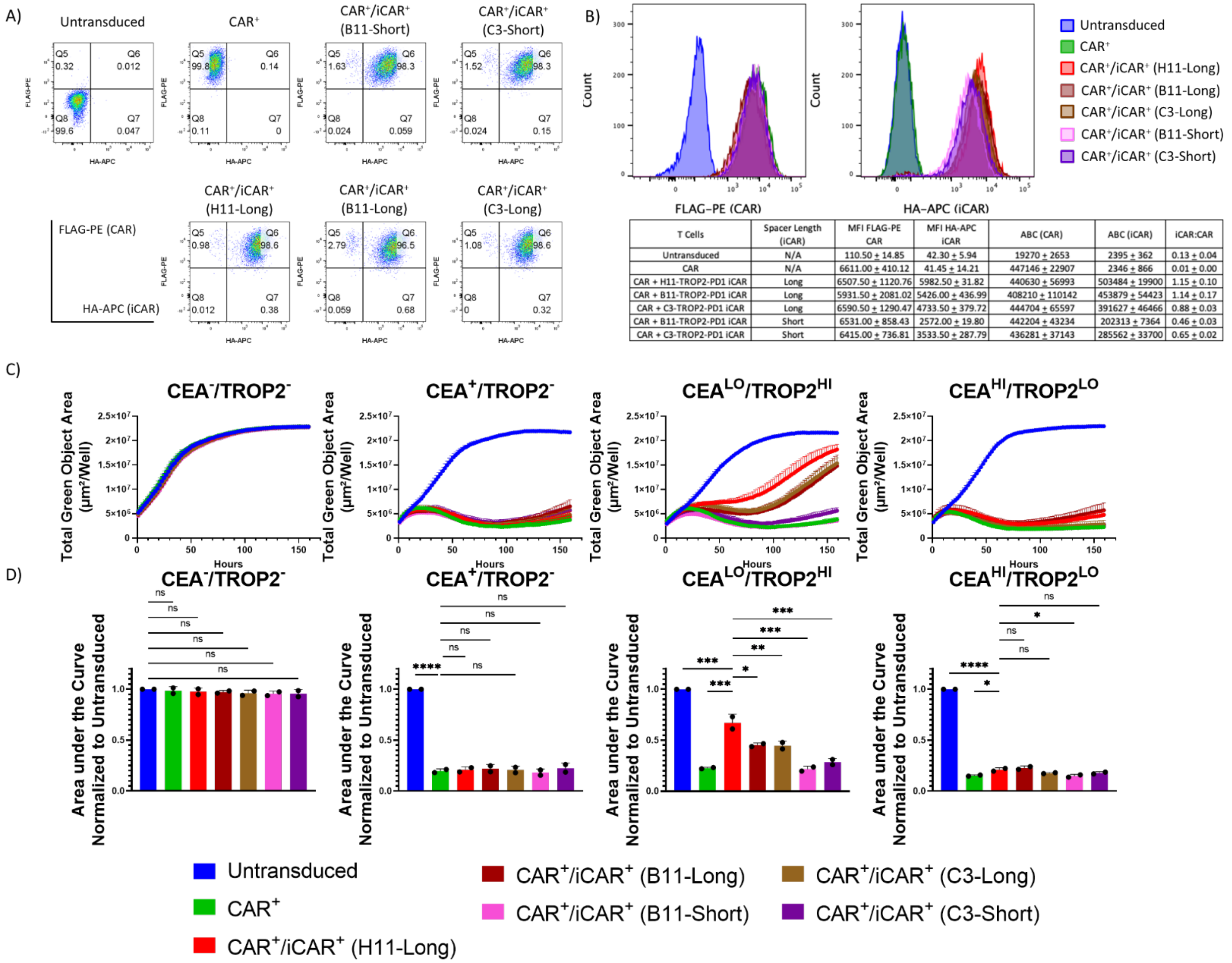

T Cells	Spacer Length (iCAR)	MFI FLAG-PE CAR	MFI HA-APC iCAR	ABC (CAR)	ABC (iCAR)	iCAR:CAR
Untransduced	N/A	110.50 ± 14.85	42.30 ± 5.94	19270 ± 2653	2395 ± 362	0.13 ± 0.04
CAR	N/A	6611.00 ± 410.12	41.45 ± 14.21	447146 ± 22907	2346 ± 866	0.01 ± 0.00
CAR + H11-TROP2-PD1 iCAR	Long	6507.50 ± 1120.76	5982.50 ± 31.82	440630 ± 56993	503484 ± 19900	1.15 ± 0.10
CAR + B11-TROP2-PD1 iCAR	Long	5931.50 ± 2081.02	5426.00 ± 436.99	408210 ± 110142	453879 ± 54423	1.14 ± 0.17
CAR + C3-TROP2-PD1 iCAR	Long	6590.50 ± 1290.47	4733.50 ± 379.72	444704 ± 65597	391627 ± 46466	0.88 ± 0.03
CAR + B11-TROP2-PD1 iCAR	Short	6531.00 ± 858.43	2572.00 ± 19.80	442204 ± 43234	202313 ± 7364	0.46 ± 0.03
CAR + C3-TROP2-PD1 iCAR	Short	6415.00 ± 736.81	3533.50 ± 287.79	436281 ± 37143	285562 ± 33700	0.65 ± 0.02

Fig. S4. TROP2-targeting iCARs with short spacers inhibit CAR-T cell cytotoxicity less efficiently than long spacer counterparts.

A) Representative flow cytometry plots confirm that CAR^+/$iCAR^+$ cells are relatively pure and express both CAR and iCAR.

B) Representative histograms of CAR and iCAR with the H11, C3, and B11 TROP2 scFv chains show stable surface expression. CAR surface expression is measured by an antibody that recognizes a FLAG-tag on the N-terminus of the CAR. iCAR surface expression is measured by an antibody that recognizes a HA-tag on the N-terminus of the iCAR. The estimated values of both CAR and iCAR on the surface of the cell were measured by quantitative flow cytometry and averaged over two independent experiments. Geometric mean fluorescence intensity of each population was measured and compared to standard curves generated by Quantum Simply Cellular beads (Bangs Laboratories, Inc.) for Anti-Mouse or Anti-Rat antibodies to extrapolate the number of CAR and iCAR molecules on the surface of the cell. Estimations were made since the MFI of the population exceeded the quantitative range of the microspheres. The iCAR:CAR ratio was calculated based on the estimates.

C) Regardless of the scFv chain used in the iCAR, TROP2-targeting iCARs with short spacers (IgG4 hinge) are less efficient at inhibiting cytotoxicity compared to their long spacer counterparts. Representative cytotoxicity curves are displayed from one experiment where the total green object area (µm/well) of GFP^{+} DU145 target cells that express CEACAM5 and/or TROP2 were measured over approximately 160 hours.

D) Area under the curve analysis of cytotoxicity curves. The delay in inhibition was measured by calculating the area under each cytotoxicity curve. The AUC was normalized against the AUC calculated for untransduced T cells co-cultured with the target cells. The normalized AUC quantified is the mean $\pm$ s.d. (n=2) from two independent experiments. The significance values shown are comparisons between a control group. For the CEA^{-}/TROP2^{-} cell line, values are compared to the untransduced control. For the CEA^{+}/TROP2^{-} cell line, values are compared to the CAR control. For the CEA^{LO}/TROP2^{HI} or CEA^{HI}/TROP2^{LO} cell lines, values are compared to the CAR^{+}/iCAR^{+} (H11-Long) group.

Statistics performed using 1 way ANOVA analysis with Tukey multiple comparison correction. *p-value $\leq$ 0.05, **p-value $\leq$ 0.01, ***p-value $\leq$ 0.001

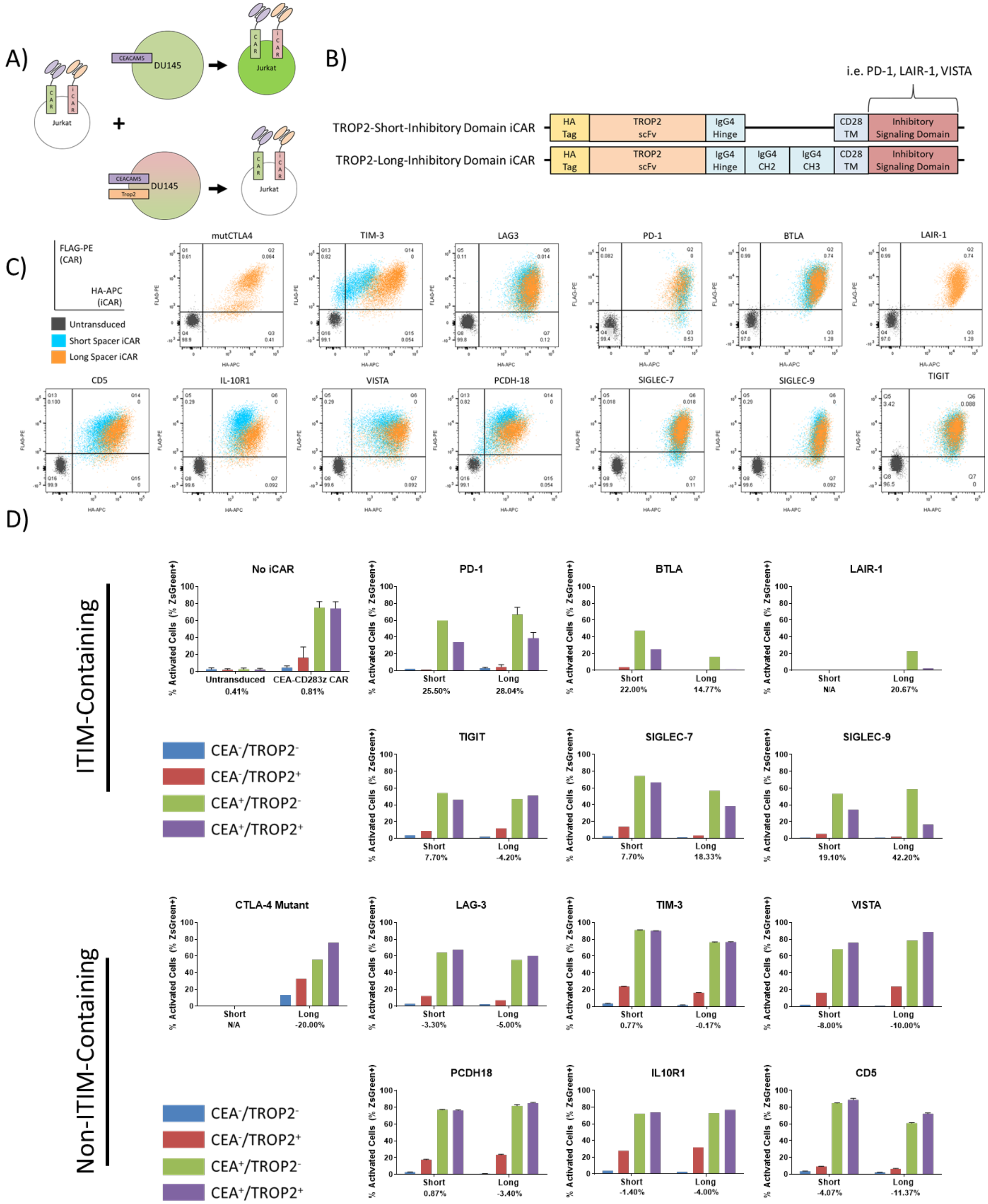

Fig. S5. ITIM-containing iCARs can inhibit CAR T cell activation in a Jurkat reporter assay.

A) An engineered Jurkat-NFAT-ZsGreen reporter cell line was transduced with the CEACAM5-Long-CD28-3z CAR and a TROP2-iCAR that contains the corresponding inhibitory signaling domain. The iCAR was transduced at a MOI of 25 and the CAR at a MOI of 1.

B) Inhibitory CAR structures are composed of a TROP2 scFv chain, a short or long spacer, a CD28 TM, and the corresponding intracellular signaling domain of the protein listed (Supplementary Table 1).

C) Jurkat-NFAT-ZsGreen cell lines transduced and sorted for $CAR^{+}/iCAR^{+}$ populations express the iCAR on the surface of the cell. Each group is compared to an untransduced control population (black). CAR and iCAR surface expression was detected using an antibody targeting the FLAG-tag or HA-tag on the construct's N-terminus respectively.

D) ITIM-containing iCARs are able to inhibit CAR T cell activation in a Jurkat-NFAT-ZsGreen reporter assay. Jurkat-NFAT-ZsGreen cell lines were transduced with CEACAM5-Long-CD28-3z CAR and one of the various TROP2-iCARs. Each cell line was sorted and co-cultured with engineered DU145 target cells at an effector:target ratio of 1:1. The percent of cells that are considered activated are measured by % $ZsGreen^{+}$ cells after gating for the CD3+ population. Percentage values under each set of graphs represent Δ Target to Non-target: Calculated difference between percent activated cells when co-cultured with target cell line ($CEA^{+}/TROP2^{-}$) versus the non-target cell line ($CEA^{+}/TROP2^{+}$). N/A were not tested.

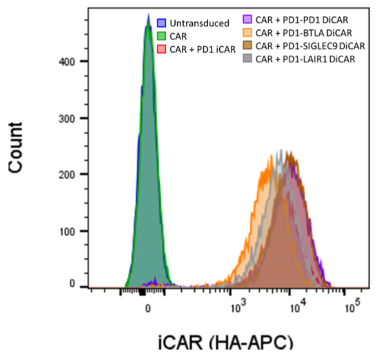

Experiment #1

T Cell	MFI (FLAG-PE) CAR	MFI (HA-APC) iCAR	ABC (CAR)	ABC (iCAR)	iCAR:CAR
Untransduced	N/A	N/A	N/A	N/A	N/A
CAR	6440	N/A	320005	N/A	N/A
CAR + PD1-iCAR	6215	6986	312088	533000	1.71
CAR + PD1-PD1 iCAR	7432	9072	353988	707200	2.00
CAR + PD1-BTLA iCAR	7958	4064	371459	296542	0.80
CAR + PD1-SIGLEC9 iCAR	8747	9080	397039	707875	1.78
CAR + PD1-LAIR1 iCAR	6755	6028	330954	454359	1.37

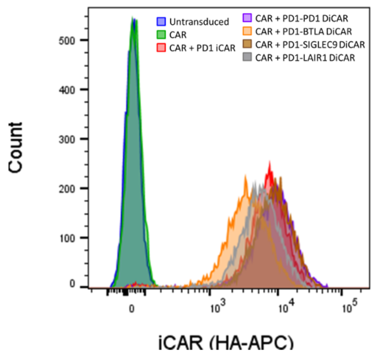

Experiment #2

T Cell	MFI (FLAG-PE) CAR	MFI (HA-APC) iCAR	ABC (CAR)	ABC (iCAR)	iCAR:CAR
Untransduced	N/A	N/A	N/A	N/A	N/A
CAR	9086	N/A	348069	N/A	N/A
CAR + PD1-iCAR	8620	6755	337249	530991	1.57
CAR + PD1-PD1 iCAR	10024	8297	369198	664037	1.80
CAR + PD1-BTLA iCAR	9286	3397	352645	251448	0.71
CAR + PD1-SIGLEC9 iCAR	10597	8133	381716	649776	1.70
CAR + PD1-LAIR1 iCAR	10145	5166	371865	396671	1.07

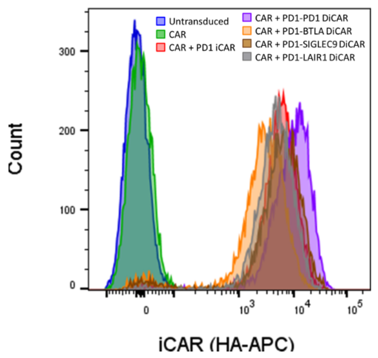

Experiment #3

T Cell	MFI (FLAG-PE) CAR	MFI (HA-APC) iCAR	ABC (CAR)	ABC (iCAR)	iCAR:CAR
Untransduced	N/A	N/A	N/A	N/A	N/A
CAR	6809	N/A	409939	N/A	N/A
CAR + PD1-iCAR	9059	5293	510309	523628	1.03
CAR + PD1-PD1 iCAR	6437	8676	392648	896803	2.28
CAR + PD1-BTLA iCAR	6511	2830	396105	264828	0.67
CAR + PD1-SIGLEC9 iCAR	6846	5675	411647	564903	1.37
CAR + PD1-LAIR1 iCAR	6469	4335	394144	421319	1.07

Fig. S6. DiCAR surface expression is similar to that of the TROP2-Long-PD1 iCAR.

Histograms of DiCARs from three independent experiments were generated to compare the surface expression of the DiCARs. Surface expression was measured by the HA-tag located on the N-terminus of each inhibitory CAR. Geometric Mean Fluorescence Intensity (MFI) was calculated for each population and reported in the tables above. The amount of CAR and iCAR (ABC – Antibody Binding Capacity) on the surface of T cells was estimated using Quantum Simply Cellular microspheres. Estimations were made since the MFI of the population exceeded the quantitative range of the microspheres. The iCAR:CAR ratio was calculated based on the estimates.

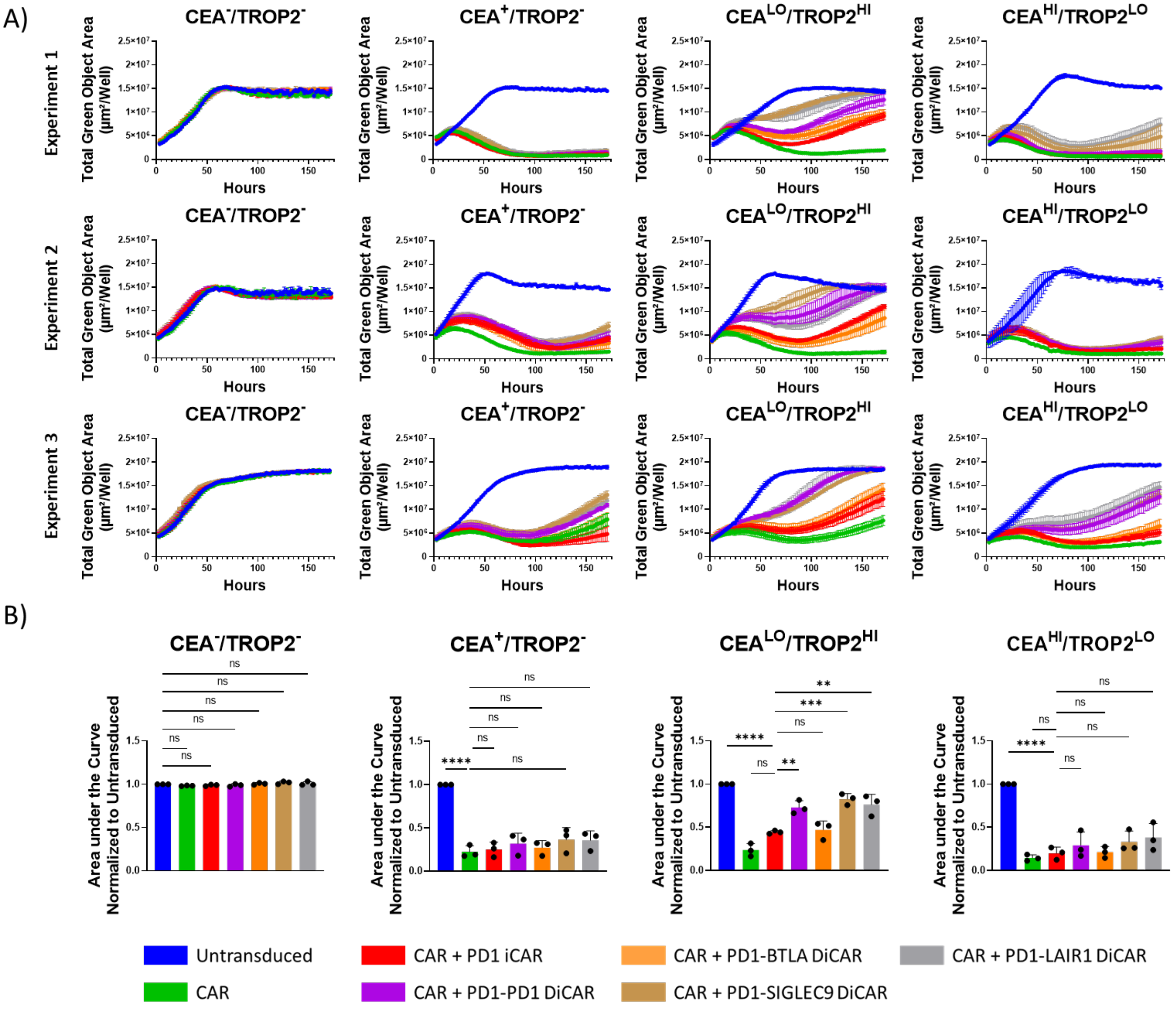

Fig. S7. Cytotoxicity curves of CAR T cells that contain a DiCAR are consistent in three experiments.

A) Cytotoxicity curves were generated by Incucyte live cell image analysis of co-cultures of T cells with GFP+ DU145 target cells that express CEACAM5 and/or TROP2. Three independent experiments were performed with three replicates using the same donor PBMCs. Presence of target cells was measured by total green object area (µm^2/well) over 150 hours.

B) The delay in inhibition of the iCAR/DiCAR was measured by area under the curve analysis of each cytotoxicity curve and normalized to the co-culture with the untransduced T cell group. The AUC displayed is an average of the three independent experiments shown above.

The significance values shown are comparisons between a control group. For the CEA$^-$/TROP2$^-$ cell line, values are compared to the Untransduced control. For the CEA$^+$/TROP2$^-$ cell

line, values are compared to the CAR control. For the CEA^{LO}/$TROP2^{HI}$ or CEA^{HI}/$TROP2^{LO}$ cell lines, values are compared to the CAR + TROP2-PD1 iCAR group.

Statistics performed using 1 way ANOVA analysis with Tukey multiple comparison correction. *p-value $\le$ 0.05, **p-value $\le$ 0.01, ***p-value $\le$ 0.001

Characteristics				Short Spacer	Long Spacer
Domain	Uniprot	Amino Acids for Intracellular Signaling Domain	ITIM Containing	Specific Inhibition Δ% ZsGreen+ ($CEA^+/TROP2^+$ - $CEA^+/TROP2^-$)	Specific Inhibition Δ% ZsGreen+ ($CEA^+/TROP2^+$ - $CEA^+/TROP2^-$)
PD1	Q15116	192-288	Yes	25.50	28.04
mutCTLA4	P16410	183-223 mutate (Y201G)	No	N/A	-20.00
BTLA	Q7Z6A9	179-289	Yes	22.00	14.77
LAIR1	Q6GTX8	187-287	Yes	N/A	20.67
TIGIT	Q495A1	163-244	Yes	7.70	-4.20
LAG3	P18627	472-525	No	-3.30	-5.00
TIM3	Q8TDQ0	224-301	No	0.77	-0.17
SIGLEC7	Q9Y286	377-467	Yes	7.70	18.33
SIGLEC9	Q9Y336	370-463	Yes	19.10	42.20
VISTA	Q9H7M9	216-311	No	-8.00	-10.00
PCDH18	D6RIG4	503-914	No	0.87	-3.40
IL10R1	Q13651	257-578 mutate (S319A, S323A, S370A)	No	-1.40	-4.00
CD5	P06127	403-495	No	-4.07	-11.37

Table S1. Structure of inhibitory CARs containing alternative inhibitory signaling domains and their ability to inhibit CAR T cell activation.

Inhibitory CARs are generated by combining the TROP2 scFv chain with a hinge/spacer, a CD28 TM, and the intracellular signaling domain of the listed protein. Each intracellular domain contains the amino acids listed defined by the Uniprot protein code. Short spacer – IgG4 Hinge; Long Spacer – IgG4 Hinge + CH2 + CH3 constant domains. Specific inhibition = The percentage difference of $ZsGreen^+$ cells between co-cultures of Jurkat cells with the $CEA^+/TROP2^+$ cell line subtracted by those co-cultured with the $CEA^+/TROP2^-$ only cell line.

Antibody	Source	Identifier
CD3 Monoclonal Antibody (SK7), APC-eFluor™ 780, eBioscience™	Invitrogen	47-0036-42
CD4 Monoclonal Antibody (OKT4 (OKT-4)), FITC, eBioscience™	Invitrogen	11-0048-42
PE/Cyanine7 anti-human CD271 (NGFR) Antibody	Biolegend	345110
PerCP/Cyanine5.5 anti-human EGFR Antibody	Biolegend	352914
PE anti-DYKDDDDK Tag Antibody	Biolegend	637310
HA Antibody, APC	Miltenyi Biotec	130-123-553
Human TROP-2 APC-conjugated Antibody	R&D Systems	FAB650A
Human CEACAM-5/CD66e Alexa Fluor® 750-conjugated Antibody	R&D Systems	FAB41281S-100UG
CD45 Monoclonal Antibody (HI30), FITC, eBioscience™	Invitrogen	11-0459-42

Table S2. Antibodies for flow cytometry staining.

SI References

1. M. Hudecek, *et al.*, The nonsignaling extracellular spacer domain of chimeric antigen receptors is decisive for in vivo antitumor activity. *Cancer Immunol Res* **3**, 125–135 (2015).
